# MRI Epicenters Differentiate Spatiotemporal Patterns of Neurodegeneration in Parkinson's Disease

**DOI:** 10.1002/advs.202511289

**Published:** 2025-09-17

**Authors:** Xiaojie Duanmu, Zihao Zhu, Jiaqi Wen, Jianmei Qin, Qianshi Zheng, Weijin Yuan, Yingni Jin, Nan Lu, Lu Wang, Cheng Zhou, Tao Guo, Haoting Wu, Chenqing Wu, Ziyi Zhu, Lifang Wang, Jingwen Chen, Jingjing Wu, Bingting Zhu, Yuelin Fang, Yaping Yan, Baorong Zhang, Minming Zhang, Xiaojun Guan, Xiaojun Xu

**Affiliations:** ^1^ Department of Radiology The Second Affiliated Hospital Zhejiang University School of Medicine Zhejiang 31009 China; ^2^ Joint Laboratory of Clinical Radiology the Second Affiliated Hospital Zhejiang University School of Medicine Hangzhou 31009 China; ^3^ Department of Neurology The Second Affiliated Hospital Zhejiang University School of Medicine Zhejiang 31009 China

**Keywords:** connectivity‐based epicenter model, deformation‐based morphometry, diffusion tensor imaging, quantitative susceptibility imaging

## Abstract

Parkinson's disease (PD) exhibits clinical and neuropathological heterogeneity, potentially driven by distinct spatiotemporal neurodegenerative patterns. This study utilizes a connectivity‐based MRI epicenter model combined with unsupervised clustering to identify unique degenerative epicenters in PD. Analyzing cross‐sectional multi‐modal MRI data from 278 PD patients and 177 healthy controls, this work identifies two distinct neurodegenerative epicenter patterns. Subtype 1 exhibits epicenters predominantly in cerebellar and midbrain regions associated with severe motor symptoms and rapid progression. Subtype 2 shows epicenters primarily in cortical and striatal regions with milder progression. These patterns are validated in an independent cohort of 66 PD patients and shows consistency in longitudinal follow‐up. Additionally, a predictive model incorporating epicenter traits and structural connectivity properties is developed, accurately forecasting individualized neurodegenerative progression. Spatial correlation analyses further reveal overlapping epicenter distributions between PD subtype 1 and other movement disorders, including essential tremor and multiple system atrophy, suggesting potential shared pathological mechanisms. These results delineate PD heterogeneity through distinct epicenter‐driven neurodegenerative trajectories, bridging the gap between neuroanatomical spread patterns and clinical variability. This novel framework not only enhances the understanding of PD's neuropathological complexity but also advances personalized prognosis and highlights connectivity‐based epicenters as promising biomarkers for PD subtyping and therapeutic targeting.

## Graphical abstract

A connectivity‐based MRI epicenter model identifies two Parkinson's disease subtypes: subtype 1 with infratentorial‐dominant epicenters, severe motor symptoms, and rapid progression; and subtype 2 with supratentorial‐dominant epicenters with mild progression. These epicenter‐driven trajectories delineate disease heterogeneity, bridging the gap between neuroanatomical spread patterns and clinical variability, and underscore connectivity‐based epicenters as biomarkers for personalized prognosis.

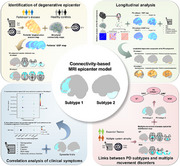

## 1 Introduction

Parkinson's disease (PD) is a prevalent progressive neurodegenerative disorder marked by significant clinical heterogeneity, including varied symptom profiles, progression rates, and outcomes,^[^ ^1^, ^2^, ^3^ ^]^ suggesting the presence of distinct subtypes. Previous efforts to classify these subtypes based on multidomain symptomatology have identified subgroups with notable differences in clinical presentation and progression.^[^ ^4^, ^5^ ^]^ However, these classifications relied heavily on subjective clinical assessments and failed to capture the underlying neuropathological complexity. Understanding PD's neuropathological heterogeneity is thus critical for elucidating its clinical variability and advancing precise therapeutic strategies.

The clinical diversity in PD is likely driven by differential spatiotemporal spread of α‐synuclein (α‐syn) pathology.^[^ ^6^, ^7^, ^8^ ^]^ The prion‐like spread of α‐syn plays a central role in PD pathogenesis, beginning in specific brain regions, where early neurodegeneration occurs, and subsequently propagating through neuronal networks.^[^ ^9^ ^]^ Current research has primarily focused on exploring universal neurodegenerative mechanisms in PD, such as Braak's hypothesis of “down‐top” spread.^[^ ^10^ ^]^ However, as clinical variability among patients becomes widely acknowledged, existing single patterns struggle to explain this heterogeneity.^[^ ^11^, ^12^ ^]^ A novel dual‐origin hypothesis suggests that PD involves distinct neurodegeneration patterns selectively damaging specific neural circuits, contributing to clinical heterogeneity.^[^ ^13^, ^14^ ^]^ However, current evidence, primarily from animal models and postmortem studies, lacks in vivo data, limiting our understanding of clinico‐neurodegenerative link in PD.

Magnetic resonance imaging (MRI) has provided a non‐invasive in vivo method to quantify neurodegeneration.^[^ ^15^ ^]^ Neurodegeneration in PD, such as cortical thinning^[^ ^16^ ^]^ and iron metabolism abnormalities in deep brain nuclei (DBN),^[^ ^17^ ^]^ like substantia nigra (SN), are observed at population level. However, these differences do not occur simultaneously across all regions, exhibiting significant spatiotemporal variations.^[^ ^16^, ^17^ ^]^ A recent epicenter theory based on intrinsic brain connectivity may explains this variation: neurodegeneration may initiate in a specific brain region, termed “epicenter,” then spreads “transneuronally” to other regions facilitated by the underlying patterns of connectivity.^[^ ^18^, ^19^ ^]^ This neuroimaging‐derived, connectivity‐based epicenter model has been successful identified potential disease onset sites and accurately predicted neurophysiological progression in dementia^[^ ^18^ ^]^ and schizophrenia.^[^ ^20^ ^]^ Applying it to PD may further elucidate the onset site and transneuronal spread of degeneration. Furthermore, MRI studies have revealed significant variations in neurodegenerative processes across PD subtypes, no matter in macro volume,^[^ ^21^ ^]^ microstructure,^[^ ^22^ ^]^ or metabolism.^[^ ^23^ ^]^ One explanation for this variation may lie in the poor understanding of neurodegenerative epicenters, which may be the driving factor for transneuronal spread and lead to the divergent patterns. While recent data‐driven clustering studies have explored distinct patterns of neurodegeneration in PD,^[^ ^24^, ^25^, ^26^ ^]^ they have primarily focused on identifying affected brain regions, neglecting the critical role of brain connectivity—the foundation of neurodegeneration spread—thus limiting the characterization of spatiotemporal heterogeneity. Thus, this study aims to apply connectivity‐based epicenter model and unsupervised clustering to identify potential heterogenous neurodegenerative epicenters in PD, providing a foundation for further investigation into the spatiotemporal patterns of neurodegenerative heterogeneity.

In addition to being evident among PD patients, the clinical heterogeneity of PD is frequently confused by other movement disorders, suggesting a shared but unclear pathological mechanism between PD and these disorders: for instance, tremor may be seen in both PD and essential tremor (ET),^[^ ^27^ ^]^ while various motor and non‐motor symptoms are shared between PD and multiple system atrophy (MSA).^[^ ^28^ ^]^ Analyzing the correlation of the neurodegenerative epicenter distribution between PD and other movement disorders may reveal shared neuropathological mechanisms underlying the overlapping clinical symptoms.

In summary, the central hypothesis of this study posits that PD involves distinct neurodegenerative epicenters, driving heterogeneous disease trajectories through unique transneuronal spread mechanisms. Three analyses were conducted to test this hypothesis: First, connectivity‐based epicenter model was applied to cross‐sectional data to construct patient‐tailored epicenter maps, analyzed via unsupervised clustering^[^ ^29^ ^]^ to identify PD subtype‐specific patterns, and validated in an independent cohort. Second, a predictive model was developed in longitudinal subcohort, incorporating individual‐specific epicenter and structural network traits to predict patient‐specific degeneration progression and elucidate subtype‐specific spatiotemporal trajectories. Finally, spin‐corrected spatial correlation analysis explored shared epicenter distributions between PD subtypes and other movement disorders (ET and MSA) to investigate potential neuro pathological commonalities.

## 2 Results

### 2.1 Experimental Design

**Figure 1:**
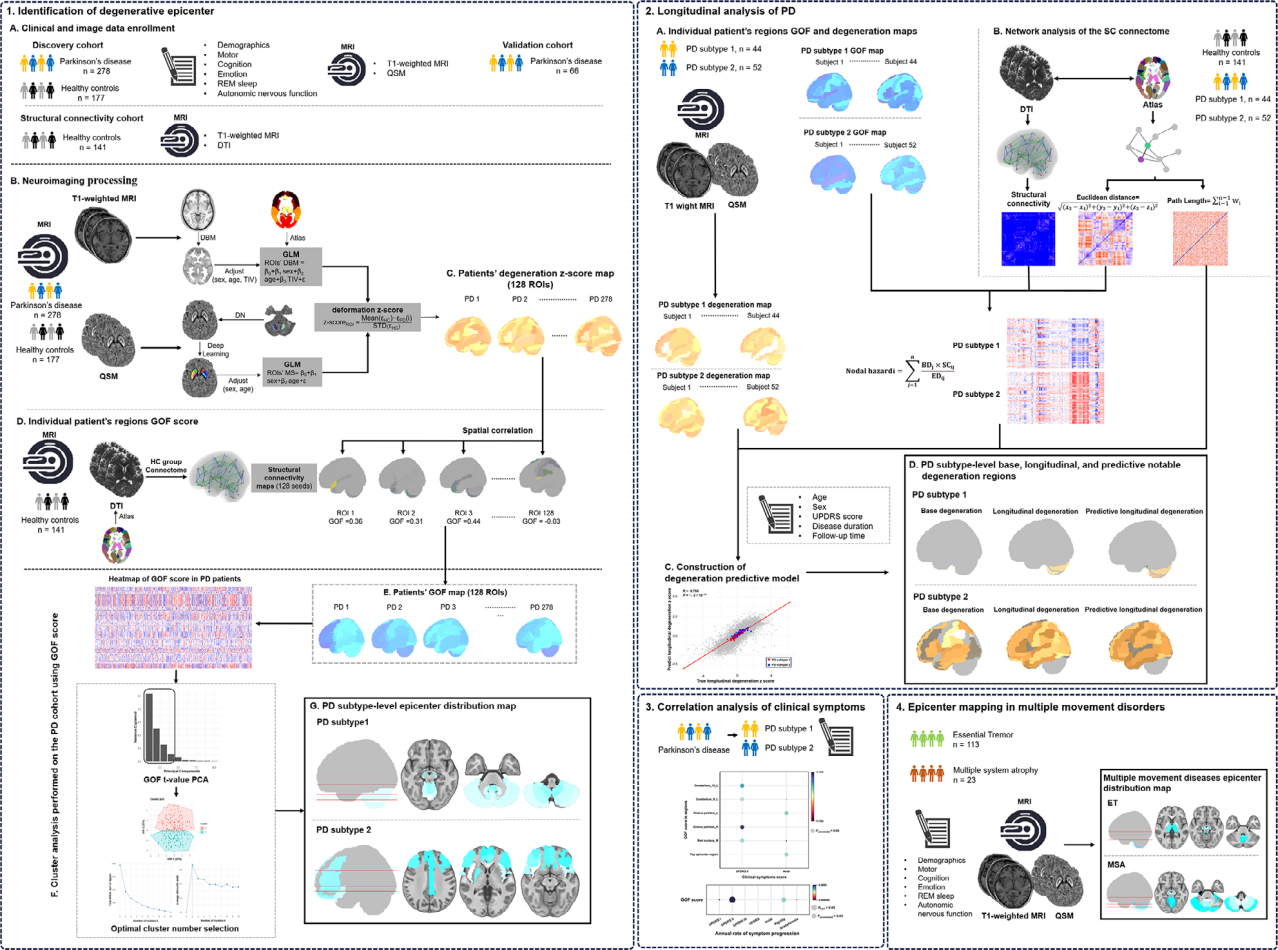
Experimental design and methodology. (1‐1 Identification of degenerative epicenter) A) 521 participants (455 discovery, 66 validation) were analyzed using six clinical data types and three MRI modalities. B) GM volume deformation (DBM) or magnetic susceptibility (QSM) were analyzed, adjusting for sex, age, and TIV (in DBM). PD patients’ scores were standardized using z‐score relative to HC. C–E) Spatial correlation between PD degeneration z‐score maps and SC maps from HC identified degenerative epicenter. Spearman correlation quantified GOF scores, generating GOP map for each patient. F) GOF score matrix underwent K‐means clustering with PCA noise reduction. The NbClust package determined optimal clusters. G) One‐sample t‐tests identified significant epicenter regions, producing t‐maps for PD subtypes. (1‐2 Longitudinal analysis of PD) A) Baseline and longitudinal data of 96 PD patients (44 subtype 1, and 52 subtype 2) were collected. Degeneration z‐score maps and baseline GOF maps were obtained for each patient. B) Euclidean distance, shortest path length, and nodal hazard were calculated by integrating individual baseline degeneration map of PD, SC map of HC, and the intrinsic spatial properties between ROIs. C,D) A predictive model for degeneration was constructed using neuroimaging features and baseline clinical data to predict longitudinal degeneration. (1‐3 Correlation analysis of clinical symptoms) Partial correlation analysis evaluated GOF score‐symptom relationships in epicenter regions, controlling for sex, age, and disease duration; and evaluated baseline GOF score‐annual symptom progression rates relationships in top epicenter region, controlling for sex, baseline age, and disease duration. (1‐4 Epicenter mapping in multiple movement diseases) Consistent analytical procedures identified the epicenter patterns of ET and MSA, with spin‐corrected spatial correlation analysis comparing these to PD subtypes. DBM = deformation‐based morphometry; DN = dentate nucleus; DTI = diffusion tensor imaging; ET = essential tremor; FDR = false discovery rate; GLM = general linear models; GM = gray matter; GOF = goodness of fit; HC = healthy controls; MRI = magnetic resonance imaging; MSA = multiple system atrophy; PCA = principal component analysis; PD = Parkinson's disease; QSM = quantitative susceptibility mapping; ROI = region of interest; SC = structural connectivity; TIV = Total intracranial volume.

**Figure** **1** provides a flowchart of experimental design. First, patient‐tailored degenerative epicenter mapping was performed using a connectivity‐based epicenter model algorithm, based on cross‐sectional T1‐weighted MRI and quantitative susceptibility mapping (QSM) from 278 PD patients in discovery cohort (PD_DC_, **Table** **1**). To address PD heterogeneity, K‐means clustering classified PD subtypes based on distinct epicenter maps, revealing subtype‐specific epicenter distribution patterns. Second, to validate epicenter as a reliable biomarker, exploratory analyses included: (i) assessing reproducibility of epicenter patterns in a longitudinal subcohort of 96 PD patients from PD_DC_ (Table 3) and an independent validation cohort of 66 PD patients (PD_VC_, Table 1); (ii) mapping PD clinical symptoms to epicenter degree; and (iii) integrating epicenter traits with structural network properties to develop a predictive model for individualized longitudinal neurodegenerative progression. Third, epicenter patterns were compared across PD and 136 patients with ET and MSA (Table S2, Supporting Information), using spin‐test corrected spatial correlation analysis to explore shared neurodegenerative mechanisms.

**Table 1:** Group demographics and clinical status in HC, PD_DC_, and PD_VC_.

	HC (177)	PD_DC_ (278)	PD_VC_ (66)	*P_1_*	*P_2_*	*P_3_*	*P_4_*
**Sex (M/F)**	74/103	145/133	32/31	**0.023**	0.094	0.358	1.000
**Age, y, mean ± SD**	60.38 ± 7.76	59.73 ± 9.68	59.34 ± 8.90	0.381	1.000	1.000	1.000
**MMSE scores, mean ± SD**	28.18 ± 2.01	26.00 ± 4.58	26.25 ± 3.67	**<0.001**	**<0.001**	**<0.001**	1.000
**HAMD scores, mean ± SD**	2.55 ± 3.14	6.18 ± 6.07	7.31 ± 6.12	**<0.001**	**<0.001**	**<0.001**	0.371
**HAMA scores, mean ± SD**	3.19 ± 3.70	5.60 ± 5.05	6.75 ± 6.51	**<0.001**	**<0.001**	**<0.001**	0.264
**SCOPA‐AUT scores, mean ± SD**	–	8.73 ± 5.82	8.33 ± 7.14	–	–	–	0.669
**RBDQ‐HK scores, mean ± SD**	–	18.01 ± 14.38	14.53 ± 13.19	–	–	–	0.088
**Disease duration, y, mean ± SD**	–	3.75 ± 3.99	3.00 ± 3.51	–	–	–	0.162
**H‐Y stages, mean ± SD**	–	2.20 ± 0.57	2.11 ± 0.42	–	–	–	0.213
**UPDRS scores, mean ± SD**	–	34.19 ± 19.98	34.82 ± 16.06	–	–	–	0.813
**TIV, mm^3^, mean ± SD**	1458.74 ± 137.77	1481.73 ± 149.36	1454.27 ± 129.17	0.182	0.286	1.000	0.485

HC = healthy controls; PD_DC_ = Parkinson's disease discovery cohort; PD_VC_ = Parkinson's disease validation cohort; MMSE = the Mini‐Mental State Examination; HAMD = Hamilton Depression Scale; HAMA = Hamilton Anxiety Scale; SCOPA‐AUT = the Scales for Outcomes in Parkinson's disease‐Autonomic; RBDQ = Rapid Eye Movement Sleep Behavior Disorder Questionnaire—Chinese University of Hong Kong version; H‐Y stages = Hoehn–Yahr stage; UPDRS = the Unified Parkinson's Disease Rating Scale; TIV = Total intracranial volume.

*P_1_*: HC vs PD, *P_2_*: HC vs PD_DC_, *P_3_*: HC vs PD_VC_, *P_4_*: PD_DC_ vs PD_VC_. **Bold,** *P* < 0.05, significant difference between groups.

### 2.2 Diverse Degenerative Epicenters of PD Subtypes

Using cross‐sectional MRI data, we derived patient‐tailored degenerative epicenter map (see Methods), quantifying epicenter goodness of fit (GOF) scores for each region as an epicenter across the whole‐brain. The GOF score reflects the spatial consistency between this region's whole‐brain structural connectivity (SC) map and patient‐specific degeneration map, with higher GOF scores indicating a greater likelihood of that region being an epicenter at individual level. Unsupervised clustering analysis of GOF scores from 128 brain regions across all PD_DC_ patients identified two subtypes (n_subtype 1_ = 142, n_subtype 2_ = 136), with K‐means identifying two as the optimal cluster number, supported by NbClust (12 votes) and an average silhouette score of 0.33 (Figure S2, Supporting Information). Demographic, clinical, and neuropsychological data are provided in **Table** **2**. Patients in PD subtype 1 exhibited more severe clinical symptoms than those in subtype 2, particularly in Unified Parkinson’s Disease Rating Scale (UPDRS) II scores (*P* = 0.025) and axial symptoms (*P* = 0.047). In addition, in the PD_DC_ cohort, a total of 134 PD patients underwent standardized dopaminergic challenge testing (n_subtype 1_ = 69, n_subtype2_ = 65). Both subtypes showed significant improvements in motor symptoms following medication, including UPDRS III, axial, tremor, rigidity, and bradykinesia scores (all *P* < 0.001). Notably, subtype 2 patients demonstrated a greater improvement in bradykinesia compared with subtype 1 (*P* = 0.037) (Table S3, Supporting Information). No significant differences were found in demographics or neuropsychological scores between the subtypes.

**Table 2:** Demographics and clinical status in PD subtypes.

	PD_DC_		PD_VC_		*P*_1_	*P*_2_	*P*_3_	*P*_4_
	Subtype 1 (142)	Subtype 2 (136)	Subtype 1 (18)	Subtype 2 (48)				
**Sex (M/F)**	80/62	65/71	10/8	25/23	0.155	0.805	0.950	0.612
**Age, y, mean ± SD**	60.01 ± 10.68	59.43 ± 8.53	50.67 ± 10.51	60.19 ± 8.18	0.615	0.206	0.271	0.590
**MMSE scores, mean ± SD**	25.68 ± 4.72	26.33 ± 4.42	25.18 ± 3.93	26.64 ± 3.54	0.234	0.161	0.676	0.667
**HAMD scores, mean ± SD**	6.40 ± 6.58	5.95 ± 5.50	7.82 ± 6.71	7.13 ± 5.96	0.535	0.689	0.402	0.214
**HAMA scores, mean ± SD**	5.66 ± 5.21	5.54 ± 4.90	7.47 ± 6.40	6.50 ± 6.59	0.846	0.601	0.189	0.292
**SCOPA‐AUT scores, mean ± SD**	8.48 ± 5.77	9.00 ± 5.91	6.82 ± 4.48	8.87 ± 7.86	0.599	0.315	0.272	0.921
**RBDQ‐HK scores, mean ± SD**	18.59 ± 14.27	17.39 ± 14.55	16.41 ± 12.42	13.85 ± 13.52	0.558	0.497	0.555	0.165
**Disease duration, y, mean ± SD**	4.00 ± 4.11	3.49 ± 3.86	3.11 ± 2.77	2.96 ± 3.78	0.281	0.878	0.372	0.415
**H‐Y stages, mean ± SD**	2.22 ± 0.59	2.18 ± 0.55	2.03 ± 0.44	2.14 ± 0.41	0.654	0.353	0.198	0.580
**UPDRS scores, mean ± SD**	36.42 ± 21.94	31.88 ± 17.49	34.78 ± 12.41	34.83 ± 17.35	0.058	0.990	0.757	0.314
**UPDRS‐I scores, mean ± SD**	1.30 ± 1.58	1.58 ± 1.65	1.72 ± 1.57	1.50 ± 1.62	0.141	0.619	0.280	0.769
**UPDRS‐II scores, mean ± SD**	9.69 ± 6.21	8.19 ± 4.73	8.94 ± 3.52	8.58 ± 5.27	**0.025**	0.789	0.619	0.633
**UPDRS‐III scores, mean ± SD**	24.63 ± 15.12	21.53 ± 12.74	23.89 ± 9.95	24.44 ± 11.95	0.066	0.863	0.841	0.169
**Axial scores, mean ± SD**	4.45 ± 3.94	3.63 ± 2.87	3.94 ± 2.56	4.21 ± 2.88	**0.047**	0.734	0.596	0.228
**Tremor scores, mean ± SD**	4.06 ± 3.57	3.91 ± 3.43	3.06 ± 2.34	4.17 ± 3.30	0.731	0.195	0.249	0.655
**Rigidity scores, mean ± SD**	5.46 ± 4.26	4.75 ± 3.96	5.94 ± 2.82	6.25 ± 3.92	0.153	0.764	0.638	**0.025**
**Bradykinesia scores, mean ± SD**	10.71 ± 7.53	9.22 ± 5.91	11.11 ± 5.23	10.37 ± 5.89	0.068	0.643	0.827	0.246
**TIV, mm^3^, mean ± SD**	1485.33 ± 158.91	1477.97 ± 139.18	1417.95 ± 117.10	1467.89 ± 131.99	0.682	0.164	0.084	0.662

PD_DC_ = Parkinson's disease discovery cohort; PD_VC_ = Parkinson's disease validation cohort; MMSE = the Mini‐Mental State Examination; HAMD = Hamilton Depression Scale; HAMA = Hamilton Anxiety Scale; SCOPA‐AUT = the Scales for Outcomes in Parkinson's disease‐Autonomic; RBDQ = Rapid Eye Movement Sleep Behavior Disorder Questionnaire—Chinese University of Hong Kong version; H‐Y stages = Hoehn–Yahr stage; UPDRS = the Unified Parkinson's Disease Rating Scale; TIV = Total intracranial volume.

*P*_1_: subtype 1 vs subtype 2 in PD_DC_; *P*_2_: subtype 1 vs subtype 2 in PD_VC_; *P*_3_: PD_DC_ subtype 1 vs PD_VC_ subtype 1; *P*_3_: PD_DC_ subtype 2 vs PD_VC_ subtype 2.

**Bold,** *P* < 0.05, significant difference between groups.

**Figure 2:**
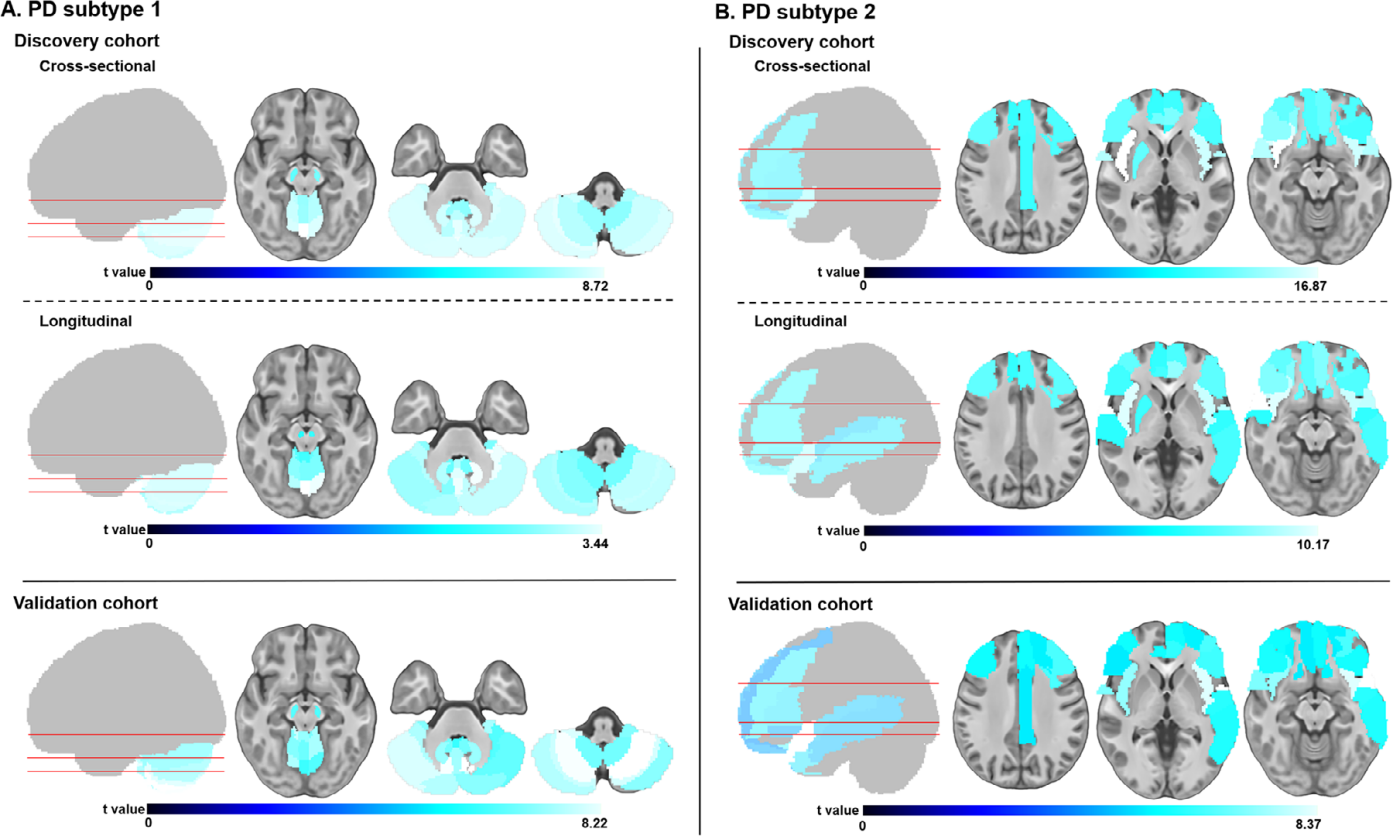
Top 30 epicenter distribution of PD subtypes. The top 30 epicenter regions for two PD subtypes (A: subtype 1, B: subtype 2) in cross‐sectional, longitudinal cohorts of PD_DC_, as well as cross‐sectional cohort of PD_VC_, defined as the top 30 regions with the highest epicenter degree after FDR correction (*p* < 0.05). The color bar of t‐values reflects the magnitude of t‐values from one‐sample t‐tests of epicenter goodness‐of‐fit score in each region, with lighter colors indicating higher t‐values and greater epicenter degree. FDR = false discovery rate; PD = Parkinson's disease; PD_DC_ = PD discovery cohort; PD_VC_ = PD validation cohort.

One‐sample *t*‐test was performed on GOF scores of brain regions across different subgroups. Regions with statistically significant t‐values (*P* < 0.05 after false discovery rate (FDR) correction), ranking in top 30, were defined as epicenter regions. The resulting t‐map revealed distinct epicenter distribution patterns for two PD subtypes at group‐level: subtype 1 demonstrated predominant epicenter involvement in cerebellar, midbrain nuclei, including cerebellum hemispheres, vermis, bilateral dentate nucleus (DN) and SN (**Figure** **2**A and Table S4, Supporting Information), which may reflect an infratentorial‐dominant (ID) distribution. Subtype 2 exhibited characteristic epicenter distribution across cerebral cortex and striatum, with higher t‐values in partial frontal, insular, and partial temporal cortices, partial cingulate gyrus and right putamen (PUT) (Figure 2B and Table S5, Supporting Information), establishing a supratentorial‐dominant (SD) distribution.

To minimize potential algorithmic bias, hierarchical clustering was applied as an auxiliary approach to validate the identified epicenter distribution patterns by K‐means clustering. As a result, hierarchical clustering also identified two PD subtypes (Figure S2, Supporting Information), and the subtype‐specific epicenter distribution patterns showed strong spatial correspondence with those obtained from K‐means clustering (subtype 1: r = 0.835; subtype 2: r = 0.794, both *P*_spin_ < 0.001) (Figure S3, Supporting Information). These findings indicated the robustness and reproducibility of the distinct epicenter distribution patterns characterizing the PD subtypes.

In PD_VC_, two subtypes were determined as the optimal solution (n_subtype 1_ = 18, n_subtype 2_ = 48, Table 2), as supported by K‐means clustering, 10 NbClust votes, and an average silhouette score of 0.35. Subtype‐specific epicenter t‐maps were calculated in PD_VC_, revealing significant spatial concordance in epicenter distributions of two PD subtypes between PD_DC_ and PD_VC_ (subtype 1: r = 0.985, *P*_spin_ < 0.001; subtype 2: r = 0.797, *P*_spin_ < 0.001) (Figure 2, Tables S4 and S5, Supporting Information). These findings demonstrated reproducibility and robustness of epicenter distribution in both PD subtypes. Moreover, in the longitudinal subcohort of PD_DC_ (n_subtype 1_ = 44, n_subtype 2_ = 52, **Table** **3**), the epicenter distributions remained consistent with baseline patterns (subtype 1: r = 0.944, *P*_spin_ < 0.001; subtype 2: r = 0.863, *P*_spin_ < 0.001) (Figure 2, Tables S4 and S5, Supporting Information), further supporting these epicenter regions as potential initiation sites of neurodegeneration in PD.

**Table 3:** Demographics and clinical status of PD subgroups at baseline and longitudinal follow‐up.

	PD Subtype 1 (44)			PD Subtype 2 (52)			*P*_1_	*P*_2_	*P*_3_
	Baseline	Longitudinal	Annual Progress Rate	Baseline	Longitudinal	Annual Progress Rate			
**Sex (M/F)**	26/18		–	30/22		–	–	–	–
**Age, y, mean ± SD**	59.88 ± 9.41	62.50 ± 9.50	–	57.66 ± 8.34	60.47 ± 8.47	–	**<0.001**	**<0.001**	–
**Mean follow‐up time, y, mean ± SD**	–	2.62 ± 1.60	–	–	2.81 ± 1.77	–	–	–	–
**MMSE scores, mean ± SD**	26.82 ± 3.54	27.11 ± 3.21	0.27 ± 1.38	27.21 ± 3.81	26.90 ± 2.95	0.06 ± 1.25	0.405	0.333	0.407
**HAMD scores, mean ± SD**	5.48 ± 4.75	7.09 ± 7.15	0.87 ± 3.58	4.79 ± 4.30	6.55 ± 5.70	0.56 ± 3.33	0.064	0.056	0.595
**HAMA scores, mean ± SD**	5.64 ± 4.94	5.20 ± 5.64	−0.20 ± 3.88	4.27 ± 3.89	5.37 ± 5.06	0.24 ± 2.91	0.631	0.216	0.631
**SCOPA‐AUT scores, mean ± SD**	7.86 ± 5.26	9.65 ± 5.71	0.11 ± 4.91	7.92 ± 7.23	7.90 ± 6.09	0.64 ± 2.12	0.855	0.480	0.822
**RBDQ‐HK scores, mean ± SD**	16.42 ± 14.15	21.33 ± 16.89	1.62 ± 5.62	13.41 ± 12.21	18.54 ± 15.58	0.36 ± 7.00	0.073	0.540	0.430
**Disease duration, y, mean ± SD**	3.12 ± 2.52	5.76 ± 2.60	–	2.88 ± 2.47	5.68 ± 2.87	–	**<0.001**	**<0.001**	–
**H‐Y stages, mean ± SD**	2.17 ± 0.49	2.30 ± 0.44	−0.07 ± 0.46	2.21 ± 0.61	2.13 ± 0.65	−0.19 ± 0.31	0.214	0.268	0.154
**UPDRS scores, mean ± SD**	29.93 ± 14.37	40.16 ± 16.17	2.77 ± 8.34	29.08 ± 17.94	34.54 ± 18.43	−0.38 ± 7.55	**<0.001**	**0.013**	0.056
**UPDRS‐I scores, mean ± SD**	1.32 ± 1.36	2.02 ± 1.75	−0.53 ± 0.89	1.13 ± 1.21	1.79 ± 1.94	−0.68 ± 1.02	**0.015**	**0.025**	0.448
**UPDRS‐II scores, mean ± SD**	8.36 ± 3.97	11.14 ± 5.27	0.14 ± 3.29	7.10 ± 4.42	8.69 ± 5.07	−0.57 ± 2.19	**<0.001**	**0.006**	0.210
**UPDRS‐III scores, mean ± SD**	19.55 ± 9.71	25.98 ± 10.40	4.30 ± 5.58	20.29 ± 13.33	23.31 ± 13.63	1.79 ± 6.19	**<0.001**	0.071	**0.042**
**Axial scores, mean ± SD**	3.57 ± 2.63	4.84 ± 2.48	1.02 ± 1.65	3.63 ± 2.89	4.31 ± 3.49	0.63 ± 1.58	**0.004**	0.087	0.253
**Tremor scores, mean ± SD**	3.23 ± 2.82	4.07 ± 3.55	−0.32 ± 1.36	3.02 ± 3.31	3.11 ± 3.03	−0.53 ± 1.56	0.056	0.600	0.476
**Rigidity scores, mean ± SD**	4.34 ± 3.54	6.36 ± 3.91	1.55 ± 1.79	4.54 ± 3.84	5.85 ± 4.40	1.08 ± 1.94	**<0.001**	**0.010**	0.226
**Bradykinesia scores, mean ± SD**	8.25 ± 5.19	10.70 ± 5.08	2.60 ± 3.47	9.06 ± 6.22	10.23 ± 6.57	1.27 ± 3.92	**0.003**	0.233	0.087

PD = Parkinson's disease; MMSE = the Mini‐Mental State Examination; HAMD = Hamilton Depression Scale; HAMA = Hamilton Anxiety Scale; SCOPA‐AUT = the Scales for Outcomes in Parkinson's disease‐Autonomic; RBDQ = Rapid Eye Movement Sleep Behavior Disorder Questionnaire—Chinese University of Hong Kong version; H‐Y stages = Hoehn–Yahr stage; UPDRS = the Unified Parkinson's Disease Rating Scale.

*P*_1_: PD subtype 1 baseline vs longitudinal; *P*_2_: PD subtype 2 baseline vs longitudinal; *P*_3_: annual progress rate in PD subtype 1 vs PD subtype 2. **Bold,** *P* < 0.05, significant difference between groups.

**Figure 3:**
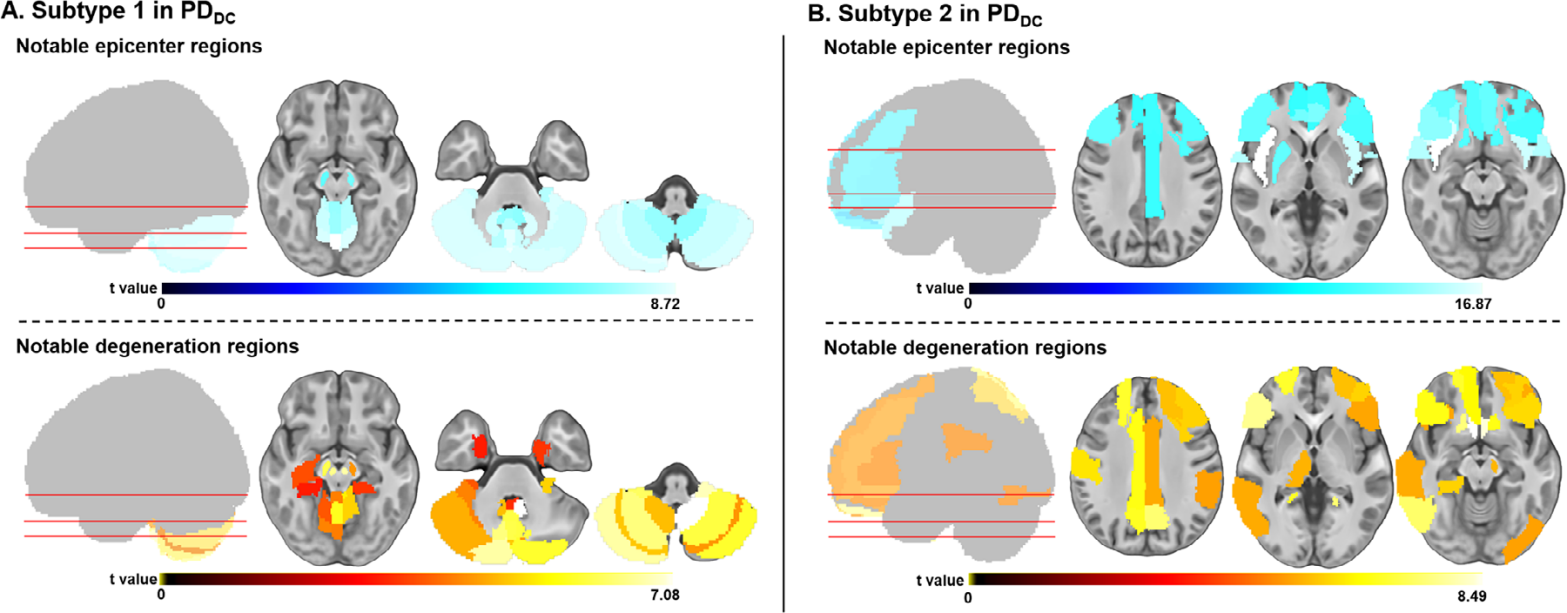
Top 30 epicenter and degeneration regions of PD subtypes in PD_DC_. The top 30 epicenter (blue) and degeneration (red) regions for two PD subtypes (A: subtype 1, B: subtype 2) in cross‐sectional cohorts of PD_DC_, defined as the top 30 regions with the highest epicenter or degeneration severity after FDR correction (*p* < 0.05). The color bar of t‐values reflects the magnitude of t‐values from one‐sample *t*‐tests of epicenter goodness‐of‐fit score or degeneration z‐score in each region, with lighter colors indicating higher t‐values and greater epicenter or degeneration severity. FDR = false discovery rate; PD = Parkinson's disease; PD_DC_ = PD discovery cohort.

Furthermore, the consistency between epicenter distribution and top 30 regions distribution of gray matter (GM) degeneration in both PD subtypes in PD_VC_ was examined (**Figure** **3**). The significant positive spatial correlations were found between epicenter mapping and degeneration mapping in both subtypes (subtype 1: r = 0.912, *P*_spin_ < 0.001; subtype 1: r = 0.391, *P*_spin_ < 0.001). The results showed that regions with higher GOF scores were generally associated with greater degeneration, suggesting consistency between epicenter and actual degeneration distribution.

### 2.3 Associations Between Regional GOF Scores and Symptoms in PD

**Figure 4:**
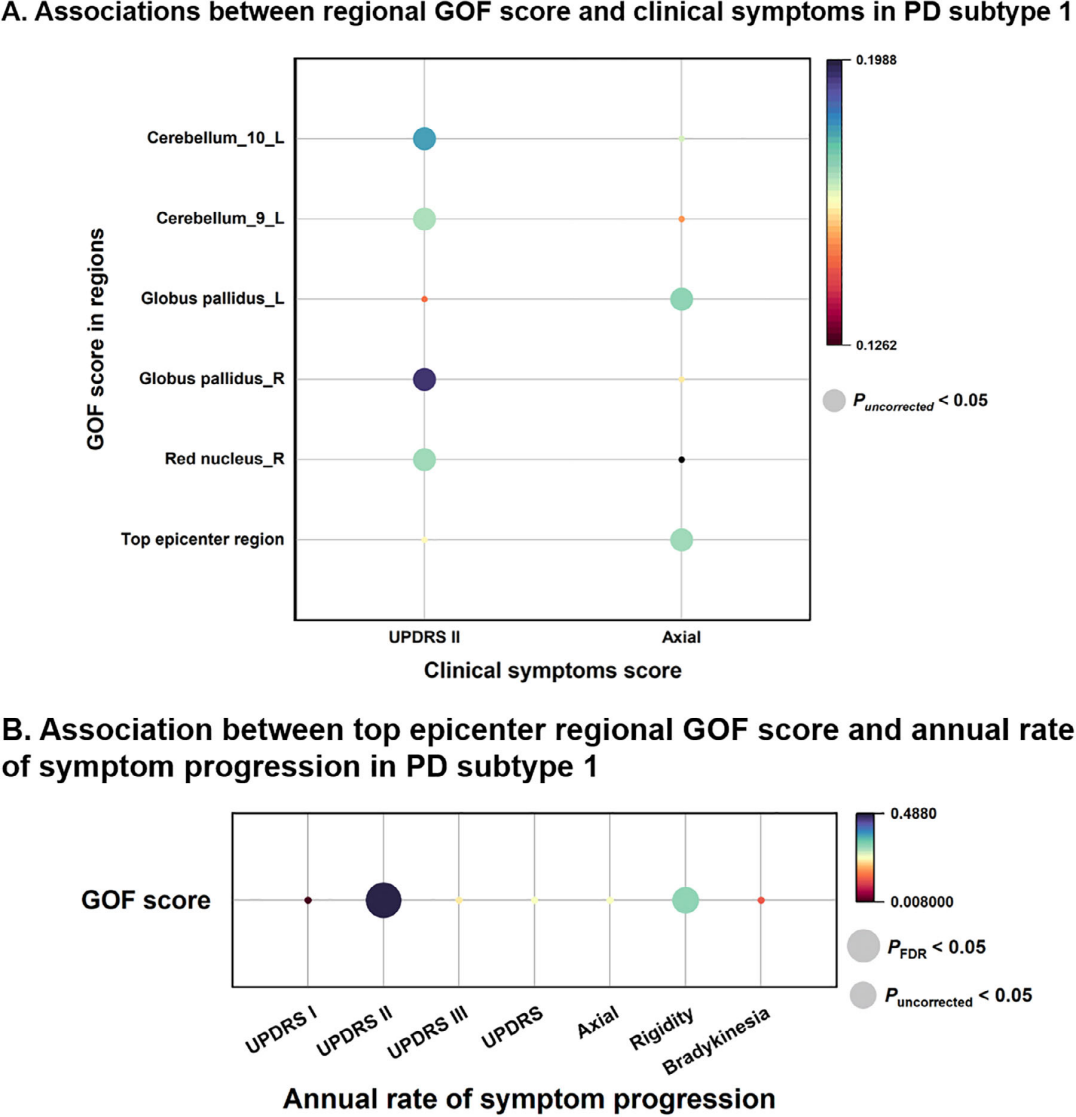
Associations between epicenter regional GOF scores and symptoms in PD. A) In PD subtype 1, GOF scores in certain epicenter regions showed a trend of positive association with UPDRS II and axial symptom scores, adjusted for sex, age, and disease duration. B) In PD subtype 1, after adjusting for sex, baseline age, and disease duration, the baseline GOF score in the top epicenter region was positively correlated with the annual progression rate of UPDRS II scores, with a potential positive correlation observed for rigidity symptoms. The circle size depicts the statistical significance level. GOF = goodness of fit; FDR = false discovery rate; L = left; PD = Parkinson's disease; R = right; UPDRS = the Unified Parkinson's Disease Rating Scale.

The associations between clinical symptoms and the GOF scores for each region (*P*_FDR_ < 0.05) were estimated (**Figure** **4**A). In PD subtype 1, trends of positive correlation with UPDRS II scores were observed in epicenter regions including left lobule IX (r = 0.169, *P*_uncorrected_ = 0.047), X (r = 0.181, *P*_uncorrected_ = 0.033) of cerebellar hemisphere, right globus pallidus (GP) (r = 0.195, *P*_uncorrected_ = 0.021), and right red nucleus (RN) (r = 0.171, *P*_uncorrected_ = 0.044). Additionally, GOF scores of left GP (r = 0.173, *P*_uncorrected_ = 0.042) and top epicenter region (r = 0.171, *P*_uncorrected_ = 0.044) showed trends of positive correlation with axial scores.

Correlations between annual rate of symptom progression and GOF scores of top epicenter region were further examined in longitudinal subcohort (Figure 4B). A significant positive correlation was found between GOF scores and UPDRS II scores (r = 0.487, *P*_FDR_ = 0.001) in PD subtype 1, with a trend of positive correlation between GOF scores and rigidity scores (r = 0.312, *P*_uncorrected_ = 0.047). The above results suggest that neurodegenerative epicenter may explain the clinical manifestations of PD.

### 2.4 Degeneration Predictive Model Predicts Longitudinal GM Degeneration in Individual Patient

A degeneration predictive model was developed in a longitudinal subcohort from PD_DC_ (n_subtype 1_ = 44, n_subtype 2_ = 52, Table 3). Demographic, clinical, and neuropsychological scores for this subcohort are provided in Table 3. During follow‐up, significant increases in total and sub‐scores of UPDRS were observed in PD subtype 1 (*P*_UPDRS_ < 0.001, *P*_UPDRS I_ = 0.015, *P*_UPDRS II_ < 0.001, *P*_UPDRS III_ < 0.001, *P*_axial_ = 0.004, *P*_rigidity_ < 0.001, *P*_bradykinesia_ = 0.003), while UPDRS I, UPDRS II, total UPDRS, and rigidity scores showed significant increase in subtype 2 (*P*_UPDRS_ = 0.013, *P*_UPDRS I_ = 0.025, *P*_UPDRS II_ = 0.006, *P*_righdity_ = 0.010). Additionally, the annual progression rate of UPDRS III scores was significantly higher in PD subtype 1 than in subtype 2 (*P* = 0.042). Overall, subtype 1 patients exhibited more severe baseline motor symptoms and faster progression than subtype 2 patients.

To effectively predict patient‐specific longitudinal degeneration and clarify spatiotemporal trajectories of neurodegeneration specific to PD subtypes, a generalized additive model (GAM) was developed using patient‐specific epicentral traits, SC properties, and clinical data. A single‐epicenter model, selected for its superior predictive efficiency, was employed^[^ ^18^ ^]^: individual‐specific top epicenter regions with the highest GOF scores were identified for each patient and used to calculate epicentral features, including Euclidean distance (ED) and shortest path length (SPE) to top epicenter. Both ED and SPE represented the distance between the top epicenter region and any given region, closely related to the degree of degeneration. However, as disease progresses, the impact of distance from epicenter on degeneration risk may diminish. Instead, future degeneration risk may be influenced by disease burden within a region's direct network neighbors. To address this, nodal hazard (NH) was introduced to quantify cumulative degeneration in a region's primary structural neighbors, predicting the vulnerability of connected regions to further neurodegeneration.

**Figure 5:**
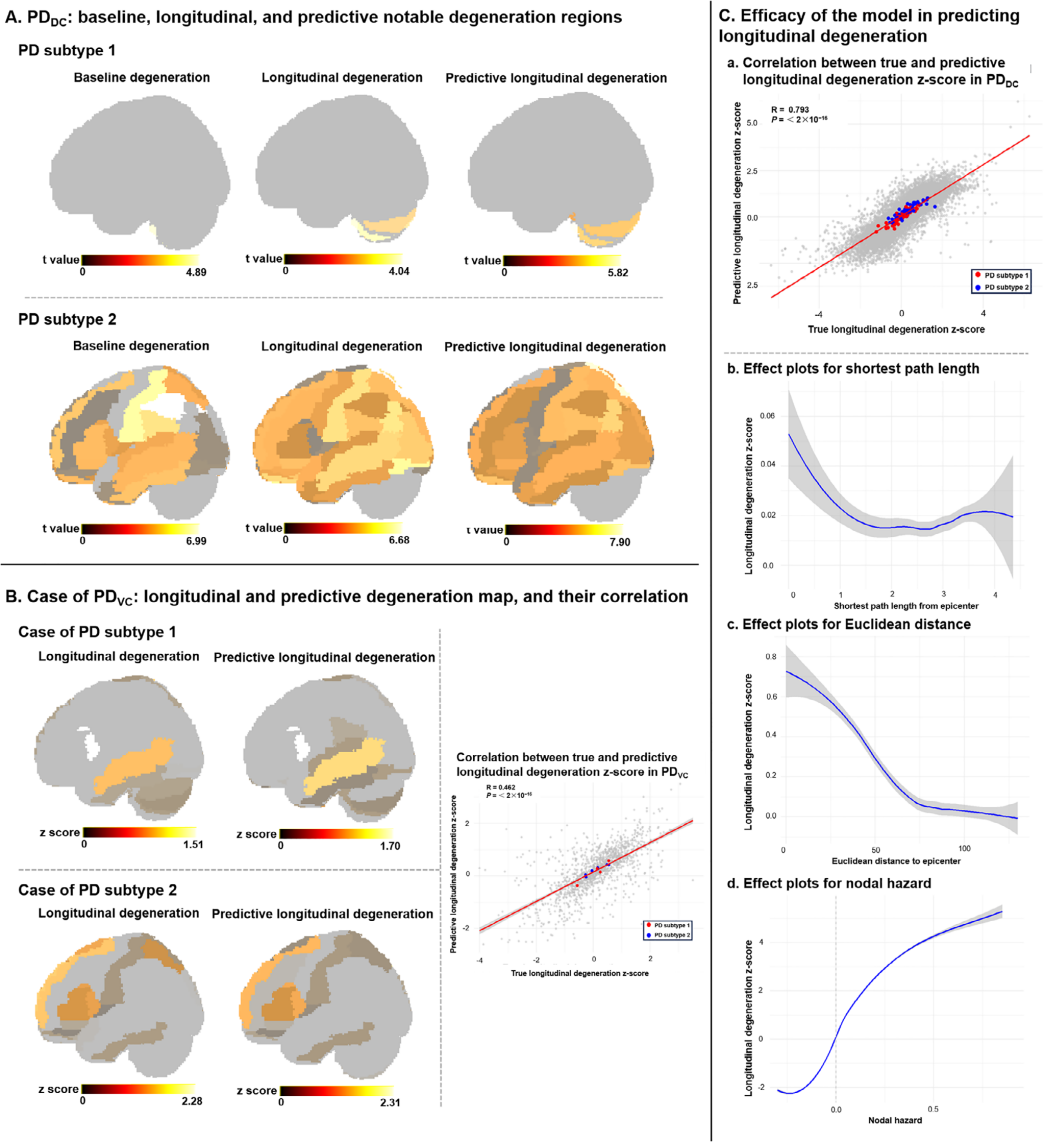
Degeneration predictive model predicting longitudinal degeneration. A) Notable degeneration regions in two PD subtypes of PD_DC_ were identified at baseline, longitudinal, and through predictive longitudinal based on model (FDR *P* < 0.05). The color bar of t‐values reflects the magnitude of t‐values from one‐sample t‐tests of degeneration z‐score in each region, with lighter colors indicating higher t‐values and degeneration severity. B) Actual and predictive longitudinal degeneration maps for one patient from each PD subtype in PD_VC_ were shown (z‐score > 0). The z‐scores color bar reflects the degeneration severity of each region, with lighter colors representing greater degeneration. Correlation analysis between true degeneration z‐score and model‐predicted score is presented. The average degeneration for each patient is depicted by larger dots, with subtype 1 in red and subtype 2 in blue. C) Correlation analysis between true degeneration z‐score in each region and model‐predicted score is presented in PD_DC_ (a). The average degeneration for each patient is depicted by larger dots, with subtype 1 in red and subtype 2 in blue. Effect plots for three predictors associated with epicenter are presented, with shaded bands c representing the 95% confidence interval: shortest path length to the top epicenter (b), Euclidean distance to the top epicenter (c), nodal hazard (d). FDR = false discovery rate; PD = Parkinson's disease; PD_DC_ = PD discovery cohort; PD_VC_ = PD validation cohort.

We evaluated the performance of model for predicting degeneration first by correlating actual and predicted longitudinal degeneration across all regions. This model explained 79.3% of regional longitudinal degeneration (**Figure** **5**C_a). Additionally, model's predicted degeneration t‐maps at subtype‐level were highly consistent with actual degeneration t‐maps (subtype 1: r = 0.930, *P*_spin_ < 0.001; subtype 2: r = 0.964, *P*_spin_ < 0.001) (Figure 5A). To further verify model reliability, independent validation was conducted on 11 patients in PD_VC_ (n_subtype 1_ = 4, n_subtype 2_ = 7) (Figure S5, Supporting Information), and the results showed that this model could effectively predict patient‐specific whole‐brain degeneration (r = 0.462, *P* < 2 × 10^−16^) (Figure 5B).

In this model, SPE (F = 6.858, *P* = 1.02 × 10^−4^), ED to epicenter (F = 31.616, *P* < 2 × 10^−16^), and NH (F = 339.050, *P* < 2 × 10^−16^) were significant predictors of longitudinal neurodegeneration (Table S7, Supporting Information). Regional‐specific SPE and ED to epicenter showed similar effects (Figure 5C_b, c): the longitudinal degeneration in top epicenter region remained significant, while degeneration in other regions decreased nonlinearly with increasing distance from epicenter, eventually plateauing. Regions with higher NH demonstrated greater longitudinal degeneration, exhibiting a nonlinear acceleration (Figure 5C_d). Additionally, baseline disease duration (F = 11.582, *P* < 2 × 10^−16^), follow‐up time (F = 53.452, *P* < 2 × 10^−16^) contributed nonlinearly to regional degeneration severity; while baseline age also had a significant positive effect on degeneration severity (t = 5.498, *P* = 3.91 × 10^−8^). Clinical symptom severity, quantified via UPDRS scores, amplified network spread through interactions with SPE (F = 7.941, *P* = 4.38 × 10^−7^) and NH (F = 5.206, *P* = 1.52 × 10^−3^), where higher baseline UPDRS scores correlated with greater global degeneration. PD subtype significantly influenced longitudinal degeneration trajectories (t = 9.507, *P* < 2 × 10^−16^), underscoring distinct progression patterns between subtypes.

### 2.5 Spatial Correlation Analysis Of Epicenter Patterns in PD and Multiple Movement Disorders

**Figure 6:**
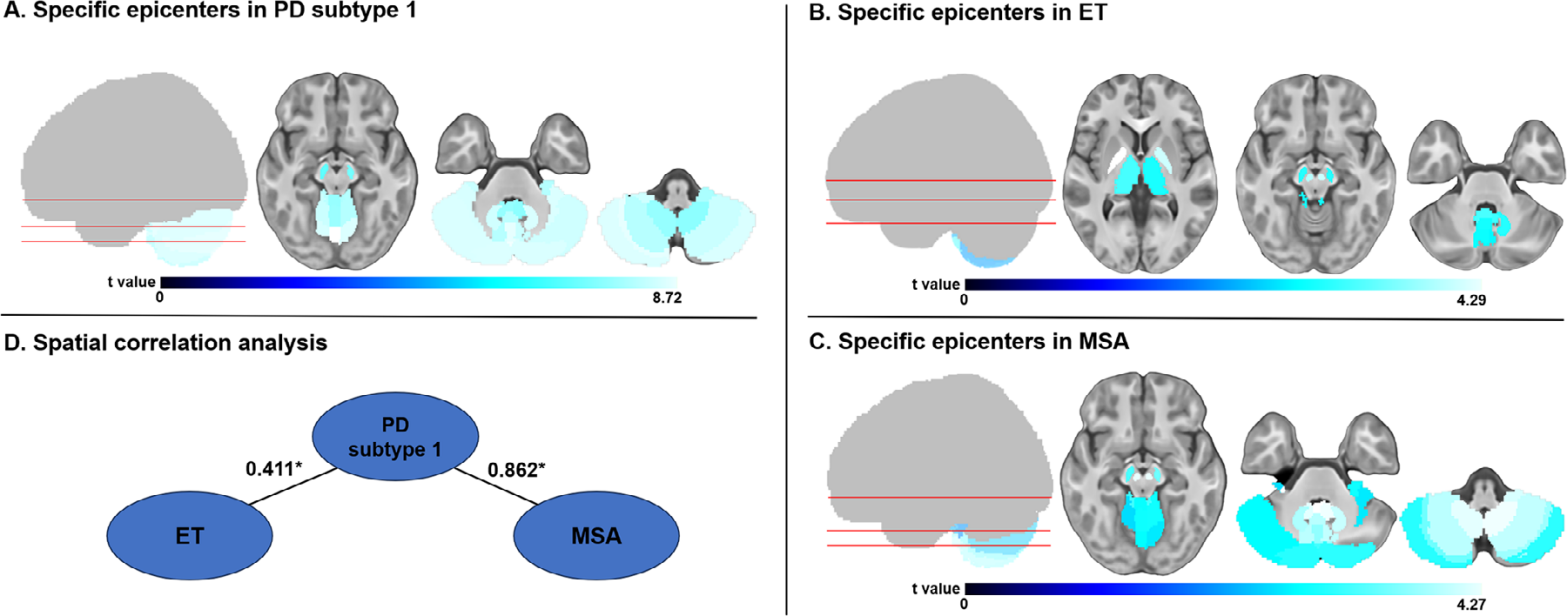
Epicenter distribution map of multiple movement disorders. A) The top 30 epicenter regions for the two PD subtypes. B) The top 30 epicenter regions for ET. C) The top 30 epicenter regions for MSA. The top 30 epicenter regions for all disorders were defined as the 30 regions with the highest epicenter degree after FDR correction (*p* < 0.05). The color bar of t‐values reflects the magnitude of t‐values from one‐sample t‐tests of epicenter goodness‐of‐fit score in each region, with lighter colors indicating higher t‐values and greater epicenter degree. D) The epicenter t‐map in PD subtype 1 was spatially correlated with the map in ET (r = 0.655, *P*_spin_ < 0.001) and MSA (r = 0.490, *P*_spin_ < 0.001) by Spearman correlation test. ET = essential tremor; FDR = false discovery rate; MSA = multiple system atrophy; PD = Parkinson's disease.

Epicenter mapping was performed for individuals diagnosed with ET (n = 113) and MSA (n = 23), with demographic, clinical, and neuropsychological data presented in Table S2, Supporting Information. In ET group, notable epicenter regions identified after FDR correction included partial cerebellar hemispheres, partial vermis, thalamus (TH), GP, SN, RN and DN (*P*_FDR_ < 0.05) (**Figure** **6**B and Table S6, Supporting Information). For MSA group, significant epicenter regions included entire cerebellar hemispheres, entire vermis, SN, RN and DN (*P*_FDR_ < 0.05) (Figure 6C and Table S6, Supporting Information). Spatial correlation analysis showed high consistency between the PD subtype 1 epicenter t‐map and those of ET (r = 0.411, *P*_spin_ < 0.001) and MSA (r = 0.862, *P*_spin_ < 0.001) (Figure 6D), indicating similar neurodegenerative epicenter distributions, potentially pointing to a common pathological origin among these diseases.

## 3 Discussion

This study identified two distinct degeneration epicenter distribution patterns in PD using patient‐tailored connectivity‐based epicenter mapping and unsupervised clustering. Subtype 1, with epicenters predominantly in cerebellar and midbrain nuclei, was designated as the ID distribution, associated with severe motor symptoms and rapid progression; whereas subtype 2, primarily involving the cerebral cortex and striatum, was classified as the SD distribution, characterized by milder symptoms, slower progression, and greater improvement in bradykinesia after dopaminergic treatment. Second, a degeneration prediction model based on epicentral traits and SC properties was developed, successfully predicting the severity of patient‐specific whole‐brain longitudinal degeneration and further confirming the heterogeneous spatiotemporal propagation patterns of neurodegeneration in PD. Third, the epicenter distribution in PD subtype 1 resembled that of ET and MSA, with overlapping epicenters primarily in the midbrain and cerebellum, suggesting a potential common pathological origin.

PD is characterized by multisystem neurodegeneration driven by α‐syn propagation, leading to region‐specific vulnerability and clinical heterogeneity.^[^ ^30^, ^31^ ^]^ Previous studies using graph theory identified group‐level neurodegeneration epicenters in PD,^[^ ^8^ ^]^ but ignored individual variability. Although recent research has started exploring heterogeneity in PD neurodegeneration^[^ ^24^, ^25^ ^]^ but remains limited to affected regions, neglecting the critical role of intrinsic connectivity in neurodegeneration spread. In this study, we applied a connectivity‐based epicenter model to create individualized epicenter maps, identifying two distinct epicenter distributions in PD through unsupervised clustering. In both PD_DC_ and PD_VC_, subtype 1 demonstrated an ID epicenter distribution, particularly including midbrain nuclei and cerebellar structures. This spatial distribution broadly aligns with traditional Braak's hypothesis, which proposes that PD pathology originates in the brainstem and progressively spreads to adjacent neural structures.^[^ ^10^ ^]^ However, we prospectively observed widespread epicenter regions in cerebellar structures in this subtype, which were not specifically explored or incorporated in the traditional Braak staging. The cerebellum has been well‐established as susceptible to abnormal α‐syn aggregation, closely associated with PD pathogenesis, and emerging evidence suggests that cerebellar involvement may play a role in modulating motor and non‐motor circuits affected in PD.^[^ ^32^, ^33^ ^]^ Our study, based on epicenter theory, further underscored the cerebellum's critical involvement in the spatiotemporal propagation of PD neurodegeneration, suggesting an extension of the traditional framework. Thus, we propose the novel ID epicenter distribution pattern, which for the first time incorporates cerebellar involvement into the pathogenic cascade, thereby refining the current framework of spatiotemporal neuropathological spread in PD. Additionally, increased GOF scores in left cerebellar lobules IX/X, right GP and RN were associated with UPDRS II progression in subtype 1, while higher GOF scores in left GP and top epicenter region correlated with worsening axial symptoms. Elevated GOF scores in a patient's top epicenter region suggested a stronger alignment between whole‐brain neurodegeneration and the region's structural connectivity. Such heightened alignment points to a broader cascade of degeneration spreading along its pathways, potentially contributing to more severe clinical symptoms. In the longitudinal cohort, baseline GOF scores in the top epicenter region positively correlated with annual progression rates of UPDRS II and rigidity, further supporting this link. Greater neurodegeneration spread along the epicenter's SC at baseline may indicate a potential for more severe symptom progression. These correlations between epicentral traits and clinical symptoms suggest that the neurodegenerative epicenters may explain clinical manifestations in PD.

In addition to the ID epicenter distribution similar to the Braak's hypothesis, our study revealed a distinct PD subtype (subtype 2) with epicenter distribution primarily located in cortical subregions and striatum. This finding is supported by recent postmortem studies and experimental models suggesting an alternative “top‐down” pathogenic pathway, where PD pathology may originate in cortical regions before spreading to the brainstem.^[^ ^34^, ^35^ ^]^ Such a cortical‐origin pattern may correspond conceptually to the “brain‐first” hypothesis, wherein cortical initiation sites influence the spatiotemporal trajectory of pathology.^[^ ^34^, ^35^ ^]^ However, compared to previously identified cortex‐origin subtypes, the epicenter distribution of subtype 2 in this study extends beyond limbic regions like cingulate gyrus, involving multiple frontal cortex subregions. This finding parallels a recent PD study identifying a neocortical subtype with early frontal cortex atrophy,^[^ ^24^ ^]^ suggesting that frontal cortex may serve as an initiation site for neurodegeneration in some PD patients, further confirming the heterogeneity of neurodegeneration in PD. Based on the epicenter distribution identified in subtype 2, in contrast to the ID pattern of the subtype 1, we defined this distribution as SD pattern. Subtype 2 showed milder baseline motor symptoms and slower progression, suggesting that cortical initiation may delay motor symptom onset. Consistent with their milder clinical presentation, both subtypes demonstrated significant improvement in motor symptoms following dopaminergic treatment; however, this effect was more pronounced in subtype 2, particularly for bradykinesia. Collectively, compared with subtype 1, patients in subtype 2 may carry a lower pathological burden, accompanied by milder clinical manifestations and greater responsiveness to dopaminergic therapy. Supporting this, previous studies have shown that, compared to those with cortical onset, PD patients with brainstem onset typically exhibit a greater α‐syn pathology burden, more symmetrical hemispheric involvement, more severe symptoms and faster disease progression.^[^ ^13^, ^36^, ^37^ ^]^ Overall, through individualized epicenter mapping, we identified two distinct neurodegenerative origins disclosing PD heterogeneity: 1) an ID distribution with rapid clinical progression, and 2) a SD distribution linked to milder progression and greater improvement in bradykinesia after dopaminergic treatment. Both epicenter distributions demonstrated strong reproducibility and robustness across longitudinal follow‐up and in the independent validation cohort. These findings not only provide imaging evidence for disease‐origin heterogeneity in PD, but also may potentially assist in the individualized prediction of both disease progression and therapeutic response in PD, enabling more precise prognosis estimation and tailored treatment planning for each patient.

Beyond elucidating the heterogeneous neurodegenerative origins of PD in cross‐sectional data, we further explored the dynamic progression trajectories of PD neurodegeneration by developing a degeneration predictive model that integrates epicentral traits and SC properties from a longitudinal subcohort. This model demonstrated strong predictive accuracy in both PD_DC_ and PD_VC_, effectively forecasting individual degeneration trajectories during follow‐up. Key parameters included epicentral metrics: First, NH, representing the risk of subsequent regional degeneration based on cumulative burden from highly connected neighboring regions,^[^ ^18^ ^]^ emerged as a significant predictor. NH explained unique variance beyond SPE and ED to the epicenter, suggesting a “contagion” mechanism in which downstream affected nodes propagate pathology. Additionally, longitudinal degeneration increased nonlinearly with NH values, supporting a sigmoidal progression pattern consistent with prior reports of nonlinear PD advancement.^[^ ^38^ ^]^ Second, as SPE and ED values, indicating that the increased distance from epicenter would diminish the longitudinal degeneration in these regions, while sustained and pronounced degeneration persisted in epicenter regions throughout the disease course. Third, the interplay between clinical symptoms and these metrics significantly predicted regional degeneration, underscoring epicentral characteristics as pivotal predictors. These features not only define the spatial origin of neurodegeneration but also offer prognostic value for anticipating individual longitudinal trajectories. Finally, the model's performance was significantly modulated by the subtypes, indicating heterogeneous spatiotemporal trajectories of neurodegeneration in PD. To summarize, our longitudinal modeling framework indicates that PD neurodegeneration originates from distinct epicenters and propagates along specific connectivity pathways, with epicenter characteristics predicting individual progression patterns, while the observed divergence in longitudinal trajectories reflects underlying heterogeneity in neuropathological mechanisms. This epicenter‐based subtype framework may further facilitate patient‐specific forecasting of disease progression and tailored monitoring strategies.

Given the clinical similarity of PD to other movement disorders such as ET and MSA, exploring the potential overlap in epicenter distribution patterns across these conditions is essential for understanding the mechanisms of PD heterogeneity. First, we observed that the epicenter distribution of PD subtype 1 closely resembled that of MSA, with overlap primarily in midbrain and cerebellum. Both PD and MSA are classified as α‐synucleinopathies, exhibit similar motor and autonomic dysfunction.^[^ ^39^, ^40^ ^]^ Consistent with our findings, degeneration of midbrain has been established as a core pathological feature of α‐synucleinopathies,^[^ ^39^, ^40^ ^]^ while cerebellar degeneration is a hallmark of MSA^[^ ^41^ ^]^ and has recently been confirmed to be associated with PD pathology.^[^ ^32^, ^33^ ^]^ These findings suggested a potential shared pathological origin between PD and MSA. In PD and ET, we also identified partial overlap in epicenter regions, suggesting a partly shared neurodegenerative origin. Unlike the extensive overlap between MSA and PD epicenters, ET exhibited several distinct epicenter regions not shared with PD, primarily in TH, GP, and RN. These regions have been pathologically linked to tremor generation^[^ ^42^, ^43^ ^]^ and may represent ET‐specific onset sites of neurodegeneration. Collectively, these findings reveal a shared epicenter degeneration pattern between PD subtype 1 and these two movement disorders, providing neuroimaging evidence into their potential neuropathological comorbidity.

We should acknowledge several limitations in present study. First, while multi‐disease analysis was a strength, small sample sizes may limit inter‐disease comparisons, necessitating validation in larger cohorts in future. Second, although the degeneration predictive model demonstrated strong performance in the longitudinal subcohort, its validation was limited by the small sample size and the availability of only a single follow‐up timepoint. These factors may affect the robustness and generalizability of the model, underscoring the necessity of replication in larger and more heterogeneous cohorts with multiple longitudinal assessments. Moreover, all participants in our study were in the clinical stage of PD. Future studies involving prodromal PD cohorts will be crucial to advance the understanding of disease epicenters to an earlier stage. Third, due to the retrospective design, medication information was incomplete and biological data such as blood or cerebrospinal fluid were unavailable, the potential effects of medication status/selection, comorbidities, and biomarker profiles on epicenter mapping remain uncertain and warranting further investigation in future prospective studies.

In conclusion, this study confirms the spatiotemporal heterogeneity of neurodegeneration in PD and, using a connectivity‐based epicenter model, identifies two distinct neurodegenerative subtypes. Each subtype originates from specific degenerative epicenters and propagates along unique brain connections. Subtype 1 originates from the ID epicenters, being associated with severe motor symptoms, rapid progression, and exhibiting a degenerative epicenter distribution that potentially shares commonalities with other movement disorders. Subtype 2, arising from SD epicenters, is associated with milder symptoms, slower disease progression, and greater improvement in bradykinesia after dopaminergic treatment. These findings establish a mechanistic framework for understanding PD heterogeneity, bridging spatial patterns of neurodegeneration with clinical variability. This novel framework not only enhances our understanding of PD's neuropathological complexity but also advances personalized prognosis and highlights connectivity‐based epicenters as promising biomarkers for PD subtyping and therapeutic targeting.

## 4 Experimental Section

### Participants

This secondary analysis was based on data from a prospective study conducted in accordance with the ethical standards of the Declaration of Helsinki and approved by the Ethics Committee of the Second Affiliated Hospital of Zhejiang University School of Medicine (Approval Reference Numbers 2017–008, February 15, 2017; and 2013–022, August 30, 2013), with written informed consent obtained from all participants.

### Cross‐Sectional Sample

A cohort of 397 PD patients was prospectively enrolled at the Department of Neurology, the Second Affiliated Hospital of Zhejiang University School of Medicine, from August 2014 to July 2024. Additionally, 191 healthy controls (HC) were recruited from the community. PD diagnoses were made by experienced neurologists according to the United Kingdom Parkinson's Disease Society Brain Bank criteria.^[^ ^44^, ^45^ ^]^ Exclusion criteria included: (1) history of neurologic or psychiatric disorders (e.g., severe brain atrophy, brain injury, cerebrovascular disease, schizophrenia, emotional disorders); (2) antipsychotic medication history in the past 3 months; (3) severe metal dentures or motion artifact. The final cohort included 344 PD patients and 177 HC (103 females, age = 60.38 ± 7.76 years) (Figure 1: 1‐1A). PD patients were divided into discovery (PD_DC_: 278 PD, 133 females, age = 59.73 ± 9.68 years) and validation cohort (PD_VC_: 66 PD, 31 females, age = 59.34 ± 8.90 years) (Figure 1: 1‐1A) based on recruitment time (before and after September 2022) with an ≈4:1 ratio.^[^ ^46^ ^]^

### Clinical and Neuropsychological Assessments

The UPDRS, Hoehn–Yahr (HY) stages, the Scales for Outcomes in Parkinson's disease‐Autonomic (SCOPA‐AUT), and Rapid Eye Movement Sleep Behavior Disorder Questionnaire—Chinese University of Hong Kong version (RBDQ‐HK) were evaluated in PD patients. Tremor, rigidity, bradykinesia, and axial symptoms subscores were calculated as follows^[^ ^47^, ^48^ ^]^: tremor (UPDRS‐III items 20–21), rigidity (UPDRS‐III item 22), bradykinesia (UPDRS‐III items 23–26 and 31), and axial symptoms (UPDRS‐III items 18 and 27–30). All participants also underwent the Mini‐Mental State Examination (MMSE), Hamilton Depression Scale (HAMD), Hamilton Anxiety Scale (HAMA). Clinical assessments and MRI scans for PD patients were conducted during the “OFF‐medication” state (a period of at least 12 h after withholding dopaminergic medications). Additionally, motor symptoms were reevaluated in an “ON‐medication” condition in 134 patients from the PD_DC_ cohort, defined as 1 h following a single dose of immediate‐release benserazide/levodopa (50/200 mg), administered immediately after the initial clinical assessment and MRI scanning.^[^ ^49^ ^]^ Medication effects were assessed as the improvement rate of motor symptom scores before and after administration, calculated as: Symptom improvement rate = (Drug off ‐Drug on)/Drug off.

### Longitudinal Sample

In PD_DC_, 96 PD patients were followed for an average of 2.73 ± 1.68 years (Figure 1: 1‐2A), while 11 PD patients in PD_VC_ were followed for 1.20 ± 0.20 years. All participants underwent clinical assessments and MRI scans at baseline and follow‐up. Disease progression was evaluated using the annual rate of clinical symptom progression [Annual progression rate = (Longitudinal ‐Baseline)/Follow‐up time].

### Multiple Movement Disorders Samples

To investigate the shared neurodegenerative mechanisms underlying the clinical overlaps among PD and other movement disorders, this work also used clinical and MRI data from the Department of Neurology, the Second Affiliated Hospital of Zhejiang University School of Medicine, included ET (n = 113) and MSA (n = 23) (Figure 1: 1‐4). All patients met the same inclusion and exclusion criteria described above, and their diagnoses were established by an experienced neurologist, with ET defined according to the Movement Disorder Society Criteria for the Diagnosis of Essential Tremor,^[^ ^50^ ^]^ and MSA diagnosed in accordance with the Movement Disorder Society Criteria for the Diagnosis of Multiple System Atrophy.^[^ ^51^ ^]^ For each patient, all scans were performed in the “OFF state” (a period at least 12 h after withholding medications).

### MRI Data Acquisition and Analysis–Neuroimaging Acquisition

All participants were scanned with a GE Discovery MR750 3.0T MRI scanner using earplugs and foam pads to minimize noise and head motion. High‐resolution 3D T1‐weighted MRI and QSM were performed for all participants. In addition, diffusion tensor imaging (DTI) data were collected from 151 HC to construct a SC cohort (Table S1, Supporting Information and Figure 1: 1‐1A). Specific sequence parameters are provided in the Supplementary Materials.

### Deformation‐Based Morphometry of GM Regions

DBM was performed using CAT12 (version 12.7; https://neuro‐jena.github.io/cat/) to quantify brain degeneration by measuring the non‐linear change required in every voxel to register the brain to the common template (IXI555 MNI152 template).^[^ ^52^ ^]^ For details on the DBM procedure, see the Supplementary Materials.

White matter (WM) in the whole‐brain Jacobian determinant map was excluded using a GM mask to minimize WM confounding effects. GM deformation values were extracted from 116 regions across cerebral cortex, cerebellar cortex, cerebellar vermis, and TH using the automated anatomical labelling atlas 3v1 (AAL 3v1 altas, https://www.gin.cnrs.fr/en/tools/aal/), and used to construct individualized degeneration maps (Figure 1: 1‐1B).

### QSM Data Processing and Segmentation of DBN

DBN are central to PD pathology,^[^ ^31^ ^]^ but traditional T1‐weighted imaging has limited resolution in subcortical regions, especially midbrain and cerebellar nuclei. Emerging QSM technology quantifies regional magnetic susceptibility, allowing for assessment of iron deposition as a marker of neurodegeneration. It offers superior sensitivity over T1‐weighted volumetric methods in evaluating DBN degeneration,^[^ ^23^, ^53^, ^54^ ^]^ as validated in our cross‐sectional data (Figure S1, Supporting Information). Therefore, this work used QSM to independently assess DBN degeneration, as outlined below:

Phase images were processed using the Susceptibility Tensor Imaging (STI) Suite V3.0 software (Duke University) on a computer cluster,^[^ ^55^ ^]^ as described in the Supplementary Materials.

The caudate (CN), PUT, GP, SN, and RN were segmented using the deep learning‐based tool DeepQSMSeg.^[^ ^56^, ^57^ ^]^ This tool was trained using manually and semi‐automatically segmented data as ground truth.^[^ ^58^, ^59^ ^]^ And DN was manually segmented using ITK‐SNAP (www.itksnap.org) by a radiologist blinded to clinical data. Regional magnetic susceptibility for the bilateral CN, PUT, GP, SN, RN, and DN were then extracted and used to construct individualized degeneration maps (Figure 1: 1‐1B).

### DTI Data Processing and SC Analysis in HC

DTI datasets were processed using FSL denoising, eddy current, and bias correction in MRtrix 3.0 (https://www.mrtrix.org/). After preprocessing, fiber orientation distributions were computed using the Single‐Shell 3‐Tissue CSD (SS3T‐CSD, https://3tissue.github.io/doc/ss3t‐csd.html) approach with a group‐averaged multi‐tissue response function. Five‐tissue‐type segmented T1‐weighted image and anatomically constrained tractography^[^ ^60^ ^]^ were used to generate 10 million whole‐brain tractograms. Spherical‐deconvolution informed filtering of tractograms (SIFT)^[^ ^61^ ^]^ was used to filter the tracts to 1 million.

The AAL 3v1 atlas defined GM nodes across cerebral cortex, cerebellar cortex, cerebellar vermis, and TH. DBN nodes, rich in iron, were defined using the MuSus‐100 atlas based on QSM.^[^ ^62^ ^]^ Both AAL 3v1 (116 regions) and MuSus‐100 (12 regions) atlases were registered to the individual native T1 space using rigid registration and nearest‐neighbor interpolation, yielding 128 GM regions. These nodes were then registered to the individual DTI space to construct structural networks. The 128 × 128 SC matrix was created using the number of valid streamlines between region pairs, with connectivity values normalized by node volume to account for differences in region size. Finally, SC matrices from 151 HC were combined to generate a group‐level mean connectivity matrix (Figure 1: 1‐1D).

### Identification of Degenerative Epicenter Patterns in PD Subtypes–Determination of Individual Regional Degeneration Z‐Score Map

Since neurodegeneration was quantified in 116 GM regions and 12 DBN using DBM and magnetic susceptibility, respectively, this work standardized all GM regions via a z‐score procedure based on the HC group. This dimensionless normalization allowed for direct comparability of neurodegeneration measures across regions.^[^ ^25^, ^26^, ^63^ ^]^ Deformation values of 116 GM regions extracted by DBM were first corrected for sex, age, and total intracranial volume (TIV) in HC using a regression model. For each PD patient, the z‐score procedure was applied to normalize the corrected GM deformation values relative to HC,^[^ ^18^, ^20^ ^]^ applying the following formula (Figure 1: 1‐1B):

(1) $$z-\mathrm{scor}e_{\mathrm{ROI}}=\frac{\mathrm{Mean}\left(\varepsilon \mathrm{HC}\right)-\varepsilon \mathrm{PD}\left(i\right)}{\mathrm{STD}\left(\varepsilon \mathrm{HC}\right)}$$

*ε_PD(i)_* represents the residual for the *i*‐th PD patient, calculated as εPD(i) = GM*_true_ ‐ GM_predict_*, where *GM_true_* is the actual deformation value and *GM_predict_* is the predicted value from the general linear model (GLM) based on HC. *Mean(ε_HC_)* and *STD(ε_HC_)* denote the mean and standard deviation of residuals from HC. The z score quantifies the severity of regional abnormalities, with higher score indicating greater deviations from normal, reflecting more severe GM degeneration.

Magnetic susceptibility of the 12 DBN, obtained QSM, were corrected for sex and age in HC using a regression model. The z‐score was then calculated to normalize the magnetic susceptibility of the corrected DBN relative to HC, with the formula given below (Figure 1: 1‐1B):

(2) $$z-\mathrm{scor}e_{\mathrm{ROI}}=\frac{\mathrm{Mean}\;\left(\varepsilon \mathrm{HC}\right)-\varepsilon \mathrm{PD}\left(i\right)}{\mathrm{STD}\left(\varepsilon \mathrm{HC}\right)}$$

The computational procedure for DBN followed the same approach as described above for GM regions, with an important modification to ensure consistency in z‐score direction across modalities. Specifically, because magnetic susceptibility values are typically higher in PD patients than in HC, the z‐score formula was adjusted to *DBN_predict_ ‐ DBN_true_*, aligning the direction of DBN z‐scores with that of DBM‐derived measures. This adjustment ensures that higher z‐scores consistently reflect greater regional abnormality across both modalities. In this framework, the z‐score quantifies the severity of regional abnormalities, with higher values indicating more severe degeneration of the DBN as measured by QSM.

Finally, the individual‐level degeneration z‐score map was generated by combining the z‐score across the 128 GM regions (Figure 1: 1‐1C).

### Determination of Individual GM Regional GOF Score

To identify the potential epicenter of neuroanatomical abnormalities in PD, this work performed patient‐specific connectivity‐based epicenter mapping. This work hypothesized that the epicenter would be a region with severe GM degeneration, whose intrinsic connectivity pattern most closely resembled the patient's GM degeneration pattern.^[^ ^16^, ^18^ ^]^ First, the SC matrix of the HC group was divided into 128 whole‐brain SC maps, using different GM regions as seed points. These maps were then spatially correlated with each patient's GM degeneration z‐score map to identify candidate epicenter regions. Spearman correlation coefficient was calculated and transformed into Fisher's z score to quantify the regional GOF score. This process produced GOF scores corresponding to 128 candidate epicenter regions, with higher GOF score indicating a greater likelihood of being the degenerative epicenter (Figure 1: 1‐1E).

### Identification of Degenerative Epicenter in PD Subtypes

The GOF dataset of 128 GM regions underwent K‐means clustering analysis (Figure 1) using R 4.4.2 (http://www.r‐project.org). To reduce background noise and enhance clustering, principal component analysis (PCA) was applied to the GOF score matrix (278 PD patients × 128 GM regions), selecting the first four principal components (PCs) that explained over 90% of the variance. In hierarchical clustering, ED was calculated between patient pairs, and Ward's linkage method was used to iteratively merge clusters while minimizing squared errors from the cluster mean.^[^ ^64^, ^65^ ^]^ The optimal number of clusters was determined using the NbClust package (Figure 1: 1‐1F). And in order to avoid the algorithm bias, this work also conducted hierarchical clustering to validate the clustering results.

For each PD subtype, one‐sample *t*‐tests of GOF scores were conducted to assess the significance of candidate epicenter regions, with the resulting t‐map reflecting the degree of epicenters across 128 regions. After multiple comparison correction, the top 30 regions with significant t‐values were selected and defined as subtype‐specific epicenter regions (Figure 1: 1‐1G). Additionally, one‐sample *t*‐tests of degeneration z‐scores at subtype level generated t‐maps of degeneration across 128 regions. Multiple comparisons were corrected using FDR.

### Links Between PD Subtypes And Multiple Movement Disorders Epicenter Maps

To investigate potential overlap in degeneration epicenter patterns between PD and other movement disorders, this work applied a consistent analysis procedure to identify epicenter patterns in ET and MSA. The spin‐corrected spatial correlation was conducted between the epicenter patterns of these disorders and those of PD subtypes, with FDR correction for multiple comparisons (Figure 1: 1‐4).

### Construction of Degeneration Predictive Model

This work developed a GAM to estimate individualized longitudinal degeneration z‐score for each region in patients, using the mgcv package^[^ ^66^ ^]^ in R 4.4.2 (http://www.r‐project.org). Based on superior prediction efficiency, this work selected single‐epicenter model over multi‐epicenter model for our analysis^[^ ^18^ ^]^: before GAM modeling, this work identified individual‐specific top epicenter regions with the highest GOF scores for each patient, which were then used for subsequent epicentral feature calculations. GAM incorporated linear terms, random effects for intercepts and slopes, and non‐linear smooth terms fitted using spline basis functions with a penalty to minimize the second derivative and maximize smoothness (Figure 1: 1‐2). Non‐linear fixed‐effect predictors included SPE, ED to the top epicenter, NH, baseline disease duration, follow‐up time, baseline UPDRS scores and its interaction with SPE and NH. Details for calculating SPE, ED, and NH are provided in the Supplementary Materials. Non‐linear basis was limited to 5 by setting k = 5.^[^ ^67^ ^]^ Linear fixed effects included baseline age, sex, PD subtypes, and the binary variable that identified a node as cortical or subcortical. Two random effects were incorporated: a random intercept for subjects and a random slope for regions. The model included a spatial autocorrelation term to account for the non‐independence of neighboring regional measurements, based on ED between each regional center of gravity coordinates (k = 30).

### Reproducibility in PD_VC_

In order to validate reproducibility, this work applied the same image processing, cluster analysis, and statistical methods to 66 PD patients in PD_VC_. Patients were successfully classified into subtypes, and GOF t‐maps were generated, resulting in identification of subtype‐specific epicenter regions. The spin‐corrected spatial correlation coefficients assessed the consistency of epicenter patterns between the PD_DC_ and PD_VC_.

To further evaluate the predictive validity of predictive model for longitudinal degeneration, baseline clinical and neuroimaging data from 11 follow‐up patients in PD_VC_ were incorporated into the model to generate patient‐specific degeneration prediction map. Predictive accuracy was quantified by calculating region‐wise correlations between actual and predictive degrees of degeneration across all regions.

### Statistical Analysis

All statistical analyses were conducted by IBM SPSS Statistics v.27.0 (Armonk, NY) and R 4.4.2.

### Demographic Data and Clinical Symptom Scores across Populations

Demographic differences among the three cohorts (PD_DC_, PD_VC_, and HC) were analyzed using chi‐square tests for sex and analysis of variance for age, MMSE scores, HAMD scores, HAMA scores, and TIV. Furthermore, two‐sample *t*‐tests were used to compare inter‐cohort differences in UPDRS scores, SCOPA‐AUT scores, RBDQ‐HK scores, H‐Y stage, and disease duration between two PD cohorts.

In both PD cohorts, chi‐square tests assessed gender differences between PD subtypes, while two‐sample t‐tests compared age, MMSE scores, HAMD scores, HAMA scores, TIV, UPDRS scores, SCOPA‐AUT scores, RBDQ‐HK scores, H‐Y stage, and disease duration between subtypes. Additionally, two‐sample t‐tests were conducted on the same subtypes across different PD cohorts for the aforementioned scores. For PD patients who received dopaminergic challenge testing, paired t‐tests evaluated changes in motor symptoms before and after medication, and a GLM was applied to examine differences in medication‐induced symptom improvement rates among PD subtypes.

In the longitudinal subcohort of PD_DC_, paired t‐tests were used to assess the differences in baseline and longitudinal clinical symptoms in PD patients. Additionally, the annual progression rate of clinical symptoms in the PD subtypes was assessed using the GLM, adjusting for sex, baseline age, and baseline disease duration.

### Associations Between Regional GOF Score and Symptoms in PD

To investigate the relationship between GOF score in epicenter regions and specific symptoms of PD subtypes, partial correlation analysis was performed between regional GOF scores and subtype‐specific clinical symptom scores, controlling for sex, age, and disease duration. Furthermore, this work explored the relationship between baseline GOF scores in the top epicenter region and the annual rate of clinical symptom progression, controlling for sex, baseline age, and disease duration. FDR correction was applied for multiple comparisons (Figure 1: 1‐3).

### Ethics Approval Statement

Our study was approved by the Medical Ethics Committee of the Second Affiliated Hospital of Zhejiang University School of Medicine, which consisted of 17 members; and all of them approved our experimental ethics.

### Patient Consent Statement

All patients signed an informed consent approved by the Medical Ethics Committee of the Second Affiliated Hospital of Zhejiang University School of Medicine.

## Conflict of Interest

The authors declare no conflict of interest.

## Author Contributions

X.D., X.G. and X.X. developed the idea of the research. X.D., Z.Z., J.W., J.Q., Q.Z., W.Y., Y.J., N.L., L.W., C.Z., T.G., H.W., C.W., Z.Z., L.W., J.C., J.W., B.Z. and Y.F. collected and organized the data. X.D., J.W. and J.Q. preprocessed and post‐processed of multi‐modal MRI data. X.D. and Z.Z. performed the statistical analyses and visualizations. X.D. wrote the initial draft of the manuscript. Y.Y., B.Z., M.Z., X.G. and X.X revised the manuscript. All authors reviewed the manuscript drafts, critically revised the manuscript, and approved the final manuscript.

## Supporting information



Supporting Information

## Data Availability

The data that support the findings of this study are available on request from the corresponding author. The data are not publicly available due to privacy or ethical restrictions.
