# Supplementary material for: MRI Epicenters Differentiate Spatiotemporal Patterns of Neurodegeneration in Parkinson's Disease
Source: Adv Sci (Weinh). 2025 Sep 17;12(45):e11289. doi: 10.1002/advs.202511289 (PMC12677683; doi:10.1002/advs.202511289)
Supplement: Supplementary file 1 — Supporting Information [file ADVS-12-e11289-s001.docx]

***Supporting Information***

**MRI Epicenters Differentiate Spatiotemporal Patterns of Neurodegeneration in Parkinson’s Disease**

Xiaojie Duanmu^1, 2^, Zihao Zhu^1, 2^, Jiaqi Wen^1, 2^, Jianmei Qin^1, 2^, Qianshi Zheng^1, 2^, Weijin Yuan^1, 2^, Yingni Jin^1, 2^, Nan Lu^1, 2^, Lu Wang^1, 2^, Cheng Zhou^1, 2^, Tao Guo^1, 2^, Haoting Wu^1, 2^, Chenqing Wu^1, 2^, Ziyi Zhu^1, 2^, Lifang Wang^1, 2^, Jingwen Chen^1, 2^, Jingjing Wu^1, 2^, Bingting Zhu^3^, Yuelin Fang^3^, Yaping Yan^3^, Baorong Zhang^3^, Minming Zhang^1, 2^, Xiaojun Guan^1, 2*^, Xiaojun Xu^1, 2*^

^1.^ Department of Radiology, The Second Affiliated Hospital, Zhejiang University School of Medicine, Zhejiang, China

^2.^ Joint Laboratory of Clinical Radiology, the Second Affiliated Hospital, Zhejiang University School of Medicine, Hangzhou, China

^3.^ Department of Neurology, The Second Affiliated Hospital, Zhejiang University School of Medicine, Zhejiang, China

^*^ Correspondence to: Dr. Xiaojun Xu (E-mail: [xxjmailbox@zju.edu.cn](mailto:xxjmailbox@zju.edu.cn)) and Xiaojun Guan (E-mail: xiaojunguan1102@zju.edu.cn) Department of Radiology, The Second Affiliated Hospital, Zhejiang University School of Medicine, No.88 Jiefang Road, Shangcheng District, Hangzhou, China, 31009, Phone: 86-0571-87315255, Fax: 86-0571-8735255.

**Supplemental Methods**

**Method S1. Neuroimaging Acquisition Sequence Parameters**

The fast spoiled gradient recalled sequence provided high-resolution 3D T1-weighted structural magnetic resonance imaging with the following parameters: repetition time (TR) = 8.1 msec; echo time (TE) = 3.0 msec; inversion time= 450 msec; flip angle = 11°; field of view (FOV) =256 × 256 mm²; voxel size = 1 × 1 × 1 mm³; a total of 196 layers. The enhanced T2-star weighted angiography sequence was used to reconstruct quantitative susceptibility mapping (QSM) with the following parameters: FOV = 240 × 240 mm², TR = 33.7 msec, TE1 / space / TE8 = 4.556 msec/ 3.648 msec/ 30.092 msec, matrix = 416×384, layer thickness = 2 mm, a total of 68 layers. Diffusion tensor imaging (DTI) was scanned by the spin echo-echo planar imaging sequence: 30 gradient directions; B value =0, 1000 s/m²; TR = 8000 msec; TE = 80 msec; flip angle = 90°; FOV = 256 × 256; matrix = 128 × 128; slice thickness = 2 mm; a total of 67 layers. Opposite phase DTI data were also collected to correct for susceptibility distortion.

**Method S2. QSM data processing**

The Susceptibility Tensor Imaging (STI) Suite V3.0 software (Duke University) was used to process phase images on a computer cluster^1^, as follows: 1) the phase images were unwrapped using the Laplacian approach, which relies solely on the sine and cosine functions of the phase angle^2,3^; 2) the background phase was eliminated using the V-SHARP method with the radius of the spherical kernel rising from 0.6 mm at the periphery of the brain to 25 mm towards the center of the brain^3^; 3) QSM images were calculated using the Streaking Artifact Reduction for QSM (STAR-QSM) method^4^. The mean signal of each individual brain was used as the susceptibility reference.

**Method S3. Deformation-Based Morphometry (DBM) data processing**

The DBM processing included bias correction, affine registration, unified segmentation^5^, skull stripping, parcellation, intensity transformation, partial volume estimation, and spatial normalization using DARTEL^6^. This generated whole-brain maps of Jacobian determinants, which were smoothed with a 12 mm isotropic kernel and used to measure local brain degeneration. Additionally, to account for image quality, subjects with poor quality (IQR > 3, as per the weighted average image quality grade reported by CAT) were excluded.

**Method S4. Group comparison of magnetic susceptibility and volume deformation of deep brain nuclei (DBN)**

To further assess whether magnetic susceptibility is more sensitive than volume deformation in detecting abnormal changes in DBN in Parkinson’s disease (PD), we compared magnetic susceptibility and volume deformation across 10 DBN between PD patients and healthy controls (HC). A general linear model was used, with age and gender as covariates, and total intracranial volume (TIV) as a covariate for volume deformation analysis. False discovery rate (FDR) correction was applied for multiple comparisons.

**Method S5. Spatial permutation test**

The spin test, a spatial permutation method, is widely used in neuroimaging to address spatial autocorrelation when assessing the correspondence between brain maps^7,8^. In this study, the spin test was based on angular permutations of the region indices. By performing 10,000 random rotations (spins) of the region indices, we generated a null distribution that preserves the spatial structure of the data. The *P*_spin_ values were then calculated by determining the proportion of random rotations where the absolute correlation was equal to or greater than the absolute observed correlation, within the generated null distribution.

**Method S6. Calculating shortest path length to the top epicenter (SPE), Euclidean distance (ED) to the top epicenter, nodal hazard (NH)**

First, the top epicenter region for each PD patient was identified for subsequent calculation of SPE, ED to epicenter, and NH.

Path length was calculated by summing the weights of edges connecting two nodes in a weighted graph, where each edge’s weight represented the cost, distance, or strength of the connection, with the following formula:

Path length = $\sum_{i-1}^{n-1} \omega_{i}$

Where *w_i_* denotes the weight of the *i*-th edge, and *n* is the total number of nodes in the path.

The ED was calculated based on the centroid coordinates of brain regions defined by atlas (automated anatomical labelling atlas 3v1, and MuSus-100 atlas) and was denoted as distance, with the following formula:

Euclidean distance =$\sqrt{\left( x_{2}-x_{2} \right)^{2}+\left( y_{2}-y_{1} \right)^{2}+\left( z_{2}-z_{1} \right)^{2}}$

*(x_1_, y_1_, z_1_)* and *(x_2_, y_2_, z_2_)* represent the centroid coordinates of two brain regions in 3D space. The squared differences between corresponding coordinates are summed and then square-rooted to obtain the straight-line distance between the regions.

NH is a novel measure that quantifies atrophy within a region’s network neighbors^9^. It represents the risk of subsequent degeneration in a region based on the cumulative degeneration burden of its most strongly connected neighbors^9^. NH was calculated by the following formula:

NH_i_ = $\sum_{j=1}^{n} \frac{\mathrm{BD}_{j}\times SC_{\mathrm{ij}}}{{ED}_{ij}}$

where *j* are the neighboring nodes of node *i*, *SC_ij_* is the edge weight between *i* and *j*, *BD_j_* is the degeneration z score for node *j*, and *ED_ij_* is the ED between *i* and *j*, and *n* = 5.

**Reference:**

1 Li, W., Avram, A. V., Wu, B., Xiao, X. & Liu, C. Integrated Laplacian-based phase unwrapping and background phase removal for quantitative susceptibility mapping. *NMR in biomedicine* **27**, 219-227, doi:10.1002/nbm.3056 (2014).

2 Li, W., Wu, B. & Liu, C. Quantitative susceptibility mapping of human brain reflects spatial variation in tissue composition. *NeuroImage* **55**, 1645-1656, doi:10.1016/j.neuroimage.2010.11.088 (2011).

3 Wu, B., Li, W., Guidon, A. & Liu, C. Whole brain susceptibility mapping using compressed sensing. *Magnetic resonance in medicine* **67**, 137-147, doi:10.1002/mrm.23000 (2012).

4 Wei, H. *et al.* Joint 2D and 3D phase processing for quantitative susceptibility mapping: application to 2D echo-planar imaging. *NMR in biomedicine* **30**, doi:10.1002/nbm.3501 (2017).

5 Ashburner, J. & Friston, K. J. Unified segmentation. *NeuroImage* **26**, 839-851, doi:10.1016/j.neuroimage.2005.02.018 (2005).

6 Ashburner, J. A fast diffeomorphic image registration algorithm. *NeuroImage* **38**, 95-113, doi:10.1016/j.neuroimage.2007.07.007 (2007).

7 Alexander-Bloch, A. F. *et al.* On testing for spatial correspondence between maps of human brain structure and function. *NeuroImage* **178**, 540-551, doi:10.1016/j.neuroimage.2018.05.070 (2018).

8 Váša, F. *et al.* Adolescent Tuning of Association Cortex in Human Structural Brain Networks. *Cerebral cortex (New York, N.Y. : 1991)* **28**, 281-294, doi:10.1093/cercor/bhx249 (2018).

9 Brown, J. A. *et al.* Patient-Tailored, Connectivity-Based Forecasts of Spreading Brain Atrophy. *Neuron* **104**, 856-868.e855, doi:10.1016/j.neuron.2019.08.037 (2019).

# Supplemental Tables

**Table S1** Demographics and clinical status in structural connectivity dataset

|  | **HC (141)** |
| --- | --- |
| **Sex (M/F)** | 57/84 |
| **Age, y, mean ± SD** | 62.17±7.84 |
| **MMSE scores, mean ± SD** | 28.09±1.96 |
| **HAMD scores, mean ± SD** | 2.81±3.84 |
| **HAMA scores, mean ± SD** | 3.31±3.99 |
| HC = healthy controls; MMSE = the Mini-Mental State Examination; HAMD = Hamilton Depression Scale; HAMA = Hamilton Anxiety Scale. | |

**Table S2** Demographics and clinical status in multiple movement disorders

|  | **ET (113)** | **MSA (23)** |
| --- | --- | --- |
| **Sex (M/F)** | 59/54 | 12/11 |
| **Age, y, mean ± SD** | 55.89±9.51 | 60.49±7.48 |
| **MMSE scores, mean ± SD** | 27.77±2.79 | 25.22±3.66 |
| **HAMD scores, mean ± SD** | 3.14±3.67 | 8.05±6.83 |
| **HAMA scores, mean ± SD** | 3.46±4.39 | 6.55±5.41 |
| **SCOPA-AUT scores, mean ± SD** | 4.05±3.92 | 16.04±9.25 |
| **RBDQ-HK scores, mean ± SD** | 8.66±8.00 | 26.61±15.80 |
| **Disease duration, y, mean ± SD** | 14.09±9.26 | 2.19±1.58 |
| **UMSARS I+II scores, mean ± SD** | - | 26.20±12.09 |
| **TETRAS scores, mean ± SD** | 27.45±12.71 | - |
| **TIV, mm^3^, mean ± SD** | 1489.66±158.68 | 1445.05±138.84 |
| ET = essential tremor; MSA = multiple system atrophy; MMSE = the Mini-Mental State Examination; HAMD = Hamilton Depression Scale; HAMA = Hamilton Anxiety Scale; SCOPA-AUT = the Scales for Outcomes in Parkinson’s disease-Autonomic; RBDQ = Rapid Eye Movement Sleep Behavior Disorder Questionnaire—Chinese University of Hong Kong version; UMSARS = Unified Multiple System Atrophy Rating Scale; TETRAS =the Essential Tremor Rating Scale; TIV = Total intracranial volume. | | |

**Table S3** Motor Symptom scores pre- and post-Levodopa challenge across PD subgroups

|  | **PD subtype 1 (69)** | | | **PD subtype 2 (65)** | | | ***P*_1_** | ***P*_2_** | ***P*_3_** |
| --- | --- | --- | --- | --- | --- | --- | --- | --- | --- |
|  | **Drug off** | **Drug on** | **Symptom improvement rate** | **Drug off** | **Drug on** | **Symptom improvement rate** |  |  |  |
| **UPDRS-III scores, mean ± SD** | 22.84±15.34 | 14.97±11.57 | 0.35±0.20 | 21.14±12.33 | 13.88±9.73 | 0.36±0.19 | **<0.001** | **<0.001** | 0.968 |
| **Axial scores, mean ± SD** | 4.13±4.29 | 3.07±3.27 | 0.21±0.34 | 3.57±2.91 | 2.86±2.51 | 0.15±0.24 | **<0.001** | **<0.001** | 0.276 |
| **Tremor scores, mean ± SD** | 3.77±3.35 | 1.88±1.93 | 0.40±0.37 | 4.54±3.39 | 2.62±2.57 | 0.37±0.37 | **<0.001** | **<0.001** | 0.643 |
| **Rigidity scores, mean ± SD** | 4.67±3.91 | 3.12±3.27 | 0.35±0.39 | 4.60±4.13 | 3.06±3.35 | 0.36±0.32 | **<0.001** | **<0.001** | 0.952 |
| **Bradykinesia scores, mean ± SD** | 10.25±7.62 | 6.81±5.86 | 0.32±0.26 | 8.60±5.70 | 5.29±4.55 | 0.42±0.26 | **<0.001** | **<0.001** | **0.037** |
| PD = Parkinson’s disease; UPDRS = the Unified Parkinson’s Disease Rating Scale.  *P*_1_: PD subtype 1 drug off vs drug on; *P*_2_: PD subtype 2 drug off vs drug on; *P*_3_: Post-treatment symptom improvement rate PD subtype 1 vs PD subtype 2.  **Bold,** *P* < 0.05, significant difference between groups. | | | | | | | | | |

**Table S4** Characterization of the significant epicenter regions in PD subtype 1

| **Discovery cohort** | | | | **Longitudinal cohort** | | | | | **Validation cohort** | | | | |  |
| --- | --- | --- | --- | --- | --- | --- | --- | --- | --- | --- | --- | --- | --- | --- |
| **Hemisphere** | **Region name** | **T values** | ***P*_FDR_ values** | **Hemisphere** | **Region name** | **T values** | ***P*_FDR_ values** | **Hemisphere** | | **Region name** | **T values** | ***P*_FDR_ values** |  |  |
| Left | Crus I of cerebellar hemisphere | 8.111 | <0.001 | Left | Crus I of cerebellar hemisphere | 3.133 | 0.006 | Left | | Crus I of cerebellar hemisphere | 6.388 | <0.001 |  |  |
| Right | Crus I of cerebellar hemisphere | 8.247 | <0.001 | Right | Crus I of cerebellar hemisphere | 2.954 | 0.008 | Right | | Crus I of cerebellar hemisphere | 7.765 | <0.001 |  |  |
| Left | Crus II of cerebellar hemisphere | 8.256 | <0.001 | Left | Crus II of cerebellar hemisphere | 3.136 | 0.006 | Left | | Crus II of cerebellar hemisphere | 6.834 | <0.001 |  |  |
| Right | Crus II of cerebellar hemisphere | 8.273 | <0.001 | Right | Crus II of cerebellar hemisphere | 3.021 | 0.007 | Right | | Crus II of cerebellar hemisphere | 7.337 | <0.001 |  |  |
| Left | Dentate nucleus | 8.235 | <0.001 | Left | Dentate nucleus | 3.382 | 0.004 | Left | | Dentate nucleus | 7.032 | <0.001 |  |  |
| Right | Dentate nucleus | 8.126 | <0.001 | Right | Dentate nucleus | 3.066 | 0.006 | Right | | Dentate nucleus | 7.006 | <0.001 |  |  |
| Left | Fusiform gyrus | 2.835 | <0.001 | Left | Lobule III of cerebellar hemisphere | 2.676 | 0.014 | Left | | Lobule III of cerebellar hemisphere | 6.660 | <0.001 |  |  |
| Left | Globus pallidus | 3.597 | <0.001 | Right | Lobule III of cerebellar hemisphere | 2.604 | 0.017 | Right | | Lobule III of cerebellar hemisphere | 7.621 | <0.001 |  |  |
| Right | Globus pallidus | 2.529 | <0.001 | Left | Lobule IV, V of cerebellar hemisphere | 2.788 | 0.012 | Left | | Lobule IV, V of cerebellar hemisphere | 7.206 | <0.001 |  |  |
| Left | Hippocampus | 2.703 | <0.001 | Right | Lobule IV, V of cerebellar hemisphere | 2.864 | 0.010 | Right | | Lobule IV, V of cerebellar hemisphere | 6.768 | <0.001 |  |  |
| Left | Lobule III of cerebellar hemisphere | 7.713 | <0.001 | Left | Lobule VI of cerebellar hemisphere | 3.348 | 0.004 | Left | | Lobule VI of cerebellar hemisphere | 6.182 | <0.001 |  |  |
| Right | Lobule III of cerebellar hemisphere | 7.863 | <0.001 | Right | Lobule VI of cerebellar hemisphere | 3.068 | 0.006 | Right | | Lobule VI of cerebellar hemisphere | 7.255 | <0.001 |  |  |
| Left | Lobule IV, V of cerebellar hemisphere | 7.788 | <0.001 | Left | Lobule VIIB of cerebellar hemisphere | 3.173 | 0.005 | Left | | Lobule VIIB of cerebellar hemisphere | 7.720 | <0.001 |  |  |
| Right | Lobule IV, V of cerebellar hemisphere | 8.046 | <0.001 | Right | Lobule VIIB of cerebellar hemisphere | 3.064 | 0.006 | Right | | Lobule VIIB of cerebellar hemisphere | 7.601 | <0.001 |  |  |
| Left | Lobule VI of cerebellar hemisphere | 8.263 | <0.001 | Left | Lobule VIII of cerebellar hemisphere | 3.126 | 0.006 | Left | | Lobule VIII of cerebellar hemisphere | 8.219 | <0.001 |  |  |
| Right | Lobule VI of cerebellar hemisphere | 8.218 | <0.001 | Right | Lobule VIII of cerebellar hemisphere | 2.833 | 0.011 | Right | | Lobule VIII of cerebellar hemisphere | 8.131 | <0.001 |  |  |
| Left | Lobule VIIB of cerebellar hemisphere | 8.054 | <0.001 | Left | Lobule IX of cerebellar hemisphere | 2.744 | 0.013 | Left | | Lobule IX of cerebellar hemisphere | 6.560 | <0.001 |  |  |
| Right | Lobule VIIB of cerebellar hemisphere | 8.401 | <0.001 | Right | Lobule IX of cerebellar hemisphere | 2.679 | 0.014 | Right | | Lobule IX of cerebellar hemisphere | 6.560 | <0.001 |  |  |
| Left | Lobule VIII of cerebellar hemisphere | 8.050 | <0.001 | Left | Lobule X of cerebellar hemisphere | 2.725 | 0.013 | Left | | Lobule X of cerebellar hemisphere | 6.761 | <0.001 |  |  |
| Right | Lobule VIII of cerebellar hemisphere | 7.813 | <0.001 | Right | Lobule X of cerebellar hemisphere | 2.806 | 0.011 | Right | | Lobule X of cerebellar hemisphere | 7.776 | <0.001 |  |  |
| Left | Lobule IX of cerebellar hemisphere | 7.290 | <0.001 | - | Lobule I, II of vermis | 2.455 | 0.023 | - | | Lobule I, II of vermis | 6.614 | <0.001 |  |  |
| Right | Lobule IX of cerebellar hemisphere | 7.392 | <0.001 | - | Lobule III of vermis | 2.502 | 0.020 | - | | Lobule III of vermis | 6.985 | <0.001 |  |  |
| Left | Lobule X of cerebellar hemisphere | 7.665 | <0.001 | - | Lobule IV, V of vermis | 2.769 | 0.012 | - | | Lobule IV, V of vermis | 6.493 | <0.001 |  |  |
| Right | Lobule X of cerebellar hemisphere | 8.103 | <0.001 | - | Lobule VI of vermis | 3.437 | 0.003 | - | | Lobule VI of vermis | 5.912 | <0.001 |  |  |
| - | Lobule I, II of vermis | 7.216 | <0.001 | - | Lobule VII of vermis | 3.220 | 0.005 | - | | Lobule VII of vermis | 6.777 | <0.001 |  |  |
| - | Lobule III of vermis | 7.407 | <0.001 | - | Lobule VIII of vermis | 3.359 | 0.004 | - | | Lobule VIII of vermis | 7.126 | <0.001 |  |  |
| - | Lobule IV, V of vermis | 7.460 | <0.001 | - | Lobule IX of vermis | 3.284 | 0.004 | - | | Lobule IX of vermis | 7.107 | <0.001 |  |  |
| - | Lobule VI of vermis | 8.717 | <0.001 | - | Lobule X of vermis | 3.047 | 0.006 | - | | Lobule X of vermis | 5.854 | <0.001 |  |  |
| - | Lobule VII of vermis | 8.557 | <0.001 | Left | Red nucleus | 2.450 | 0.023 | Left | | Parahippocampal gyrus | 4.237 | 0.001 |  |  |
| - | Lobule VIII of vermis | 8.406 | <0.001 | Right | Red nucleus | 2.363 | 0.028 | Right | | Parahippocampal gyrus | 3.675 | 0.003 |  |  |
| - | Lobule IX of vermis | 7.894 | <0.001 | Left | Substantia nigra | 2.339 | 0.029 | Left | | Red nucleus | 4.435 | 0.001 |  |  |
| - | Lobule X of vermis | 7.400 | <0.001 |  |  |  |  | Right | | Red nucleus | 4.057 | 0.001 |  |  |
| Left | Parahippocampal gyrus | 6.145 | <0.001 |  |  |  |  | Left | | Substantia nigra | 6.147 | <0.001 |  |  |
| Right | Parahippocampal gyrus | 4.500 | <0.001 |  |  |  |  | Right | | Substantia nigra | 6.227 | <0.001 |  |  |
| Left | Red nucleus | 6.167 | <0.001 |  |  |  |  |  | |  |  | <0.001 |  |  |
| Right | Red nucleus | 5.711 | <0.001 |  |  |  |  |  | |  |  | <0.001 |  |  |
| Left | Substantia nigra | 6.994 | <0.001 |  |  |  |  |  | |  |  | <0.001 |  |  |
| Right | Substantia nigra | 6.719 | <0.001 |  |  |  |  |  | |  |  | <0.001 |  |  |

**Table S5** Characterization of the significant epicenter regions in PD subtype 2

| **Discovery cohort** | | | | **Longitudinal cohort** | | | | | **Validation cohort** | | | | |
| --- | --- | --- | --- | --- | --- | --- | --- | --- | --- | --- | --- | --- | --- |
| **Hemisphere** | **Region name** | **T values** | ***P*_FDR_ values** | **Hemisphere** | **Region name** | **T values** | ***P*_FDR_ values** | **Hemisphere** | | **Region name** | **T values** | ***P*_FDR_ values** |  |
| Left | Angular gyrus | 9.392 | <0.001 | Left | Angular gyrus | 5.948 | <0.001 | Left | | Angular gyrus | 5.011 | <0.001 |  |
| Right | Angular gyrus | 9.084 | <0.001 | Right | Angular gyrus | 6.348 | <0.001 | Right | | Angular gyrus | 4.441 | <0.001 |  |
| Left | Anterior cingulate cortex, pregenual | 13.690 | <0.001 | Left | Anterior cingulate cortex, pregenual | 8.583 | <0.001 | Left | | Anterior cingulate cortex, pregenual | 5.885 | <0.001 |  |
| Right | Anterior cingulate cortex, pregenual | 13.465 | <0.001 | Right | Anterior cingulate cortex, pregenual | 7.435 | <0.001 | Right | | Anterior cingulate cortex, pregenual | 5.504 | <0.001 |  |
| Left | Anterior cingulate cortex, subgenual | 12.059 | <0.001 | Left | Anterior cingulate cortex, subgenual | 7.053 | <0.001 | Left | | Anterior cingulate cortex, subgenual | 4.777 | <0.001 |  |
| Right | Anterior cingulate cortex, subgenual | 11.768 | <0.001 | Right | Anterior cingulate cortex, subgenual | 6.710 | <0.001 | Right | | Anterior cingulate cortex, subgenual | 4.556 | <0.001 |  |
| Left | Anterior cingulate cortex, supracallosal | 12.597 | <0.001 | Left | Anterior cingulate cortex, supracallosal | 7.431 | <0.001 | Left | | Anterior cingulate cortex, supracallosal | 5.158 | <0.001 |  |
| Right | Anterior cingulate cortex, supracallosal | 12.596 | <0.001 | Right | Anterior cingulate cortex, supracallosal | 6.986 | <0.001 | Right | | Anterior cingulate cortex, supracallosal | 4.611 | <0.001 |  |
| Left | Anterior orbital gyrus | 11.820 | <0.001 | Left | Anterior orbital gyrus | 7.471 | <0.001 | Left | | Anterior orbital gyrus | 5.099 | <0.001 |  |
| Right | Anterior orbital gyrus | 13.975 | <0.001 | Right | Anterior orbital gyrus | 8.353 | <0.001 | Right | | Anterior orbital gyrus | 5.218 | <0.001 |  |
| Left | Caudate | 8.319 | <0.001 | Left | Caudate | 4.770 | <0.001 | Left | | Caudate | 3.226 | 0.003 |  |
| Right | Caudate | 8.031 | <0.001 | Right | Caudate | 4.256 | <0.001 | Right | | Caudate | 2.157 | 0.043 |  |
| Left | Cuneus | 5.988 | <0.001 | Left | Cuneus | 3.596 | <0.001 | Left | | Cuneus | 3.106 | 0.004 |  |
| Right | Cuneus | 6.059 | <0.001 | Right | Cuneus | 4.199 | <0.001 | Right | | Cuneus | 2.673 | 0.013 |  |
| Left | Gyrus rectus | 12.556 | <0.001 | Left | Gyrus rectus | 7.787 | <0.001 | Left | | Gyrus rectus | 5.201 | <0.001 |  |
| Right | Gyrus rectus | 12.665 | <0.001 | Right | Gyrus rectus | 7.849 | <0.001 | Right | | Gyrus rectus | 5.094 | <0.001 |  |
| Left | Heschl’s gyrus | 5.799 | <0.001 | Left | Heschl’s gyrus | 3.198 | <0.001 | Left | | Heschl’s gyrus | 3.042 | 0.005 |  |
| Right | Heschl’s gyrus | 9.432 | <0.001 | Right | Heschl’s gyrus | 5.285 | <0.001 | Right | | Heschl’s gyrus | 4.184 | 0.000 |  |
| Right | Hippocampus | 3.352 | <0.001 | Left | Inferior frontal gyrus, opercular part | 6.209 | <0.001 | Left | | Inferior frontal gyrus, opercular part | 4.794 | <0.001 |  |
| Left | Inferior frontal gyrus, opercular part | 10.284 | <0.001 | Right | Inferior frontal gyrus, opercular part | 5.486 | <0.001 | Right | | Inferior frontal gyrus, opercular part | 4.256 | <0.001 |  |
| Right | Inferior frontal gyrus, opercular part | 10.324 | <0.001 | Left | Inferior frontal gyrus, pars orbitalis | 7.608 | <0.001 | Left | | Inferior frontal gyrus, pars orbitalis | 5.532 | <0.001 |  |
| Left | Inferior frontal gyrus, pars orbitalis | 12.276 | <0.001 | Right | Inferior frontal gyrus, pars orbitalis | 8.360 | <0.001 | Right | | Inferior frontal gyrus, pars orbitalis | 5.591 | <0.001 |  |
| Right | Inferior frontal gyrus, pars orbitalis | 14.110 | <0.001 | Left | Inferior frontal gyrus, triangular part | 7.800 | <0.001 | Left | | Inferior frontal gyrus, triangular part | 6.056 | <0.001 |  |
| Left | Inferior frontal gyrus, triangular part | 12.447 | <0.001 | Right | Inferior frontal gyrus, triangular part | 7.367 | <0.001 | Right | | Inferior frontal gyrus, triangular part | 5.072 | <0.001 |  |
| Right | Inferior frontal gyrus, triangular part | 13.050 | <0.001 | Left | Inferior parietal gyrus, excluding supramarginal and angular gyri | 6.192 | <0.001 | Left | | Inferior parietal gyrus, excluding supramarginal and angular gyri | 4.733 | <0.001 |  |
| Left | Inferior parietal gyrus, excluding supramarginal and angular gyri | 9.682 | <0.001 | Right | Inferior parietal gyrus, excluding supramarginal and angular gyri | 5.712 | <0.001 | Right | | Inferior parietal gyrus, excluding supramarginal and angular gyri | 4.457 | <0.001 |  |
| Right | Inferior parietal gyrus, excluding supramarginal and angular gyri | 9.360 | <0.001 | Left | Inferior temporal gyrus | 5.196 | <0.001 | Left | | Inferior temporal gyrus | 4.250 | <0.001 |  |
| Left | Inferior temporal gyrus | 6.854 | <0.001 | Right | Inferior temporal gyrus | 5.252 | <0.001 | Right | | Inferior temporal gyrus | 3.428 | <0.001 |  |
| Right | Inferior temporal gyrus | 6.388 | <0.001 | Left | Insula | 9.375 | <0.001 | Left | | Insula | 7.971 | <0.001 |  |
| Left | Insula | 15.693 | <0.001 | Right | Insula | 9.682 | <0.001 | Right | | Insula | 7.068 | <0.001 |  |
| Right | Insula | 16.873 | <0.001 | Left | Lateral orbital gyrus | 5.467 | <0.001 | Left | | Lateral orbital gyrus | 3.983 | <0.001 |  |
| Left | Lateral orbital gyrus | 9.290 | <0.001 | Right | Lateral orbital gyrus | 6.887 | <0.001 | Right | | Lateral orbital gyrus | 4.591 | <0.001 |  |
| Right | Lateral orbital gyrus | 12.882 | <0.001 | Left | Medial orbital gyrus | 7.921 | <0.001 | Left | | Medial orbital gyrus | 5.461 | <0.001 |  |
| Left | Medial orbital gyrus | 12.541 | <0.001 | Right | Medial orbital gyrus | 7.937 | <0.001 | Right | | Medial orbital gyrus | 5.296 | <0.001 |  |
| Right | Medial orbital gyrus | 13.356 | <0.001 | Left | Middle cingulate & paracingulate gyri | 6.978 | <0.001 | Left | | Middle cingulate & paracingulate gyri | 5.092 | <0.001 |  |
| Left | Middle cingulate & paracingulate gyri | 12.081 | <0.001 | Right | Middle cingulate & paracingulate gyri | 6.627 | <0.001 | Right | | Middle cingulate & paracingulate gyri | 4.273 | <0.001 |  |
| Right | Middle cingulate & paracingulate gyri | 11.621 | <0.001 | Left | Middle frontal gyrus | 7.802 | <0.001 | Left | | Middle frontal gyrus | 6.186 | <0.001 |  |
| Left | Middle frontal gyrus | 12.537 | <0.001 | Right | Middle frontal gyrus | 7.862 | <0.001 | Right | | Middle frontal gyrus | 5.461 | <0.001 |  |
| Right | Middle frontal gyrus | 13.052 | <0.001 | Left | Middle occipital gyrus | 3.690 | <0.001 | Left | | Middle occipital gyrus | 3.074 | 0.005 |  |
| Left | Middle occipital gyrus | 4.799 | <0.001 | Right | Middle occipital gyrus | 3.869 | <0.001 | Right | | Middle occipital gyrus | 2.297 | 0.032 |  |
| Right | Middle occipital gyrus | 4.786 | <0.001 | Left | Middle temporal gyrus | 7.474 | <0.001 | Left | | Middle temporal gyrus | 5.664 | <0.001 |  |
| Left | Middle temporal gyrus | 10.502 | <0.001 | Right | Middle temporal gyrus | 6.903 | <0.001 | Right | | Middle temporal gyrus | 4.646 | <0.001 |  |
| Right | Middle temporal gyrus | 9.523 | <0.001 | Left | Olfactory cortex | 2.938 | <0.001 | Left | | Olfactory cortex | 2.407 | 0.025 |  |
| Left | Olfactory cortex | 6.290 | <0.001 | Right | Olfactory cortex | 4.923 | <0.001 | Right | | Olfactory cortex | 3.242 | 0.003 |  |
| Right | Olfactory cortex | 9.248 | <0.001 | Left | Postcentral gyrus | 3.419 | 0.002 | Left | | Postcentral gyrus | 2.330 | 0.030 |  |
| Left | Paracentral lobule | 2.327 | <0.001 | Right | Postcentral gyrus | 2.914 | 0.006 | Right | | Postcentral gyrus | 2.123 | 0.046 |  |
| Right | Paracentral lobule | 2.632 | <0.001 | Left | Posterior cingulate gyrus | 5.099 | <0.001 | Left | | Posterior cingulate gyrus | 3.394 | 0.002 |  |
| Left | Postcentral gyrus | 5.780 | <0.001 | Right | Posterior cingulate gyrus | 4.941 | <0.001 | Right | | Posterior cingulate gyrus | 2.503 | 0.020 |  |
| Right | Postcentral gyrus | 4.934 | <0.001 | Left | Posterior orbital gyrus | 8.433 | <0.001 | Left | | Posterior orbital gyrus | 6.544 | <0.001 |  |
| Left | Posterior cingulate gyrus | 7.722 | <0.001 | Right | Posterior orbital gyrus | 9.341 | <0.001 | Right | | Posterior orbital gyrus | 6.719 | <0.001 |  |
| Right | Posterior cingulate gyrus | 6.849 | <0.001 | Left | Precentral gyrus | 3.008 | 0.005 | Left | | Precuneus | 3.524 | 0.001 |  |
| Left | Posterior orbital gyrus | 14.302 | <0.001 | Right | Precentral gyrus | 2.111 | 0.045 | Right | | Precuneus | 3.166 | 0.004 |  |
| Right | Posterior orbital gyrus | 15.987 | <0.001 | Left | Precuneus | 5.497 | <0.001 | Left | | Putamen | 4.560 | <0.001 |  |
| Left | Precentral gyrus | 4.983 | <0.001 | Right | Precuneus | 5.662 | <0.001 | Right | | Putamen | 4.104 | <0.001 |  |
| Right | Precentral gyrus | 3.947 | <0.001 | Left | Putamen | 6.657 | <0.001 | Left | | Superior frontal gyrus, dorsolateral | 5.164 | <0.001 |  |
| Left | Precuneus | 7.761 | <0.001 | Right | Putamen | 7.332 | <0.001 | Right | | Superior frontal gyrus, dorsolateral | 4.454 | <0.001 |  |
| Right | Precuneus | 7.330 | <0.001 | Left | Rolandic operculum | 5.901 | <0.001 | Left | | Superior frontal gyrus, medial | 5.479 | <0.001 |  |
| Left | Putamen | 10.746 | <0.001 | Right | Rolandic operculum | 5.994 | <0.001 | Right | | Superior frontal gyrus, medial | 4.738 | <0.001 |  |
| Right | Putamen | 13.071 | <0.001 | Left | Superior frontal gyrus, dorsolateral | 6.657 | <0.001 | Left | | Superior frontal gyrus, medial orbital | 5.465 | <0.001 |  |
| Left | Superior frontal gyrus, dorsolateral | 11.225 | <0.001 | Right | Superior frontal gyrus, dorsolateral | 7.332 | <0.001 | Right | | Superior frontal gyrus, medial orbital | 5.116 | <0.001 |  |
| Right | Superior frontal gyrus, dorsolateral | 11.229 | <0.001 | Left | Superior frontal gyrus, medial | 7.541 | <0.001 | Left | | Superior occipital gyrus | 2.263 | 0.034 |  |
| Left | Superior frontal gyrus, medial | 12.081 | <0.001 | Right | Superior frontal gyrus, medial | 7.181 | <0.001 | Left | | Superior parietal gyrus | 3.683 | 0.001 |  |
| Right | Superior frontal gyrus, medial | 11.621 | <0.001 | Left | Superior frontal gyrus, medial orbital | 8.339 | <0.001 | Right | | Superior parietal gyrus | 3.253 | 0.003 |  |
| Left | Superior frontal gyrus, medial orbital | 13.149 | <0.001 | Right | Superior frontal gyrus, medial orbital | 7.768 | <0.001 | Left | | Superior temporal gyrus | 5.779 | <0.001 |  |
| Right | Superior frontal gyrus, medial orbital | 12.644 | <0.001 | Left | Superior occipital gyrus | 2.683 | 0.011 | Right | | Superior temporal gyrus | 4.936 | <0.001 |  |
| Left | Superior occipital gyrus | 7.781 | <0.001 | Right | Superior occipital gyrus | 3.464 | 0.001 | Left | | Supplementary motor area | 2.874 | <0.001 |  |
| Right | Superior occipital gyrus | 6.672 | <0.001 | Left | Superior parietal gyrus | 5.187 | <0.001 | Left | | SupraMarginal gyrus | 4.732 | <0.001 |  |
| Left | Superior parietal gyrus | 3.923 | <0.001 | Right | Superior parietal gyrus | 4.556 | <0.001 | Right | | SupraMarginal gyrus | 4.853 | <0.001 |  |
| Right | Superior parietal gyrus | 4.319 | <0.001 | Left | Superior temporal gyrus | 7.155 | <0.001 | Left | | Rolandic operculum | 4.392 | <0.001 |  |
| Left | Superior temporal gyrus | 10.617 | <0.001 | Right | Superior temporal gyrus | 7.066 | <0.001 | Right | | Rolandic operculum | 4.389 | <0.001 |  |
| Right | Superior temporal gyrus | 10.354 | <0.001 | Left | Supplementary motor area | 4.153 | <0.001 | Left | | Temporal pole: middle temporal gyrus | 6.092 | <0.001 |  |
| Left | Supplementary motor area | 7.157 | <0.001 | Right | Supplementary motor area | 3.403 | 0.002 | Right | | Temporal pole: middle temporal gyrus | 5.095 | <0.001 |  |
| Right | Supplementary motor area | 5.884 | <0.001 | Left | SupraMarginal gyrus | 5.990 | <0.001 | Left | | Temporal pole: superior temporal gyrus | 8.369 | <0.001 |  |
| Left | SupraMarginal gyrus | 9.942 | <0.001 | Right | SupraMarginal gyrus | 6.401 | <0.001 | Right | | Temporal pole: superior temporal gyrus | 6.813 | <0.001 |  |
| Right | SupraMarginal gyrus | 10.502 | <0.001 | Left | Temporal pole: middle temporal gyrus | 6.953 | <0.001 |  | |  |  |  |  |
| Left | Temporal pole: middle temporal gyrus | 11.589 | <0.001 | Right | Temporal pole: middle temporal gyrus | 7.847 | <0.001 |  | |  |  |  |  |
| Right | Temporal pole: middle temporal gyrus | 11.240 | <0.001 | Left | Temporal pole: superior temporal gyrus | 9.265 | <0.001 |  | |  |  |  |  |
| Left | Temporal pole: superior temporal gyrus | 15.001 | <0.001 | Right | Temporal pole: superior temporal gyrus | 10.173 | <0.001 |  | |  |  |  |  |
| Right | Temporal pole: superior temporal gyrus | 15.066 | <0.001 |  |  |  |  |  | |  |  |  |  |
| Left | Thalamus | 2.268 | 0.027 |  |  |  |  |  | |  |  |  |  |

**Table S6** Characterization of the significant epicenter regions in ET, and MSA

| **Essential tremor** | | | | **Multiple system atrophy** | | | |
| --- | --- | --- | --- | --- | --- | --- | --- |
| **Hemisphere** | **Region name** | **T values** | ***P*_FDR_ values** | **Hemisphere** | **Region name** | **T values** | ***P*_FDR_ values** |
| Left | Globus pallidus | 3.976 | 0.006 | Left | Crus I of cerebellar hemisphere | 2.471 | 0.027 |
| Right | Globus pallidus | 4.287 | 0.005 | Right | Crus I of cerebellar hemisphere | 2.697 | 0.017 |
| Left | Lobule III of cerebellar hemisphere | 2.829 | 0.038 | Left | Crus II of cerebellar hemisphere | 2.969 | 0.010 |
| Right | Lobule III of cerebellar hemisphere | 2.835 | 0.038 | Right | Crus II of cerebellar hemisphere | 2.941 | 0.011 |
| Left | Lobule VIII of cerebellar hemisphere | 2.680 | 0.047 | Left | Dentate nucleus | 3.759 | 0.002 |
| Right | Lobule VIII of cerebellar hemisphere | 2.780 | 0.038 | Right | Dentate nucleus | 4.145 | 0.001 |
| Left | Lobule IX of cerebellar hemisphere | 3.091 | 0.036 | Left | Lobule III of cerebellar hemisphere | 3.174 | 0.007 |
| Right | Lobule IX of cerebellar hemisphere | 3.142 | 0.034 | Right | Lobule III of cerebellar hemisphere | 3.098 | 0.008 |
| Left | Lobule X of cerebellar hemisphere | 3.330 | 0.030 | Left | Lobule IV, V of cerebellar hemisphere | 2.797 | 0.014 |
| Right | Lobule X of cerebellar hemisphere | 2.805 | 0.038 | Right | Lobule IV, V of cerebellar hemisphere | 2.486 | 0.027 |
| - | Lobule I, II of vermis | 2.784 | 0.038 | Left | Lobule VI of cerebellar hemisphere | 2.573 | 0.022 |
| - | Lobule VIII of vermis | 3.160 | 0.034 | Right | Lobule VI of cerebellar hemisphere | 2.355 | 0.033 |
| - | Lobule IX of vermis | 2.776 | 0.038 | Left | Lobule VIIB of cerebellar hemisphere | 3.661 | 0.003 |
| - | Lobule X of vermis | 2.830 | 0.038 | Right | Lobule VIIB of cerebellar hemisphere | 3.383 | 0.005 |
| Left | Red nucleus | 3.966 | 0.006 | Left | Lobule VIII of cerebellar hemisphere | 3.890 | 0.002 |
| Right | Red nucleus | 3.835 | 0.007 | Right | Lobule VIII of cerebellar hemisphere | 3.850 | 0.002 |
| Left | Substantia nigra | 2.948 | 0.037 | Left | Lobule IX of cerebellar hemisphere | 4.173 | 0.001 |
| Right | Substantia nigra | 2.945 | 0.037 | Right | Lobule IX of cerebellar hemisphere | 4.102 | 0.001 |
| Left | Thalamus | 2.866 | 0.038 | Left | Lobule X of cerebellar hemisphere | 4.025 | 0.001 |
| Right | Thalamus | 3.147 | 0.034 | Right | Lobule X of cerebellar hemisphere | 3.840 | 0.002 |
|  |  |  |  | - | Lobule I, II of vermis | 3.654 | 0.003 |
|  |  |  |  | - | Lobule III of vermis | 2.744 | 0.016 |
|  |  |  |  | - | Lobule IV, V of vermis | 2.775 | 0.015 |
|  |  |  |  | - | Lobule VI of vermis | 2.708 | 0.017 |
|  |  |  |  | - | Lobule VII of vermis | 3.110 | 0.008 |
|  |  |  |  | - | Lobule VIII of vermis | 3.592 | 0.003 |
|  |  |  |  | - | Lobule IX of vermis | 3.833 | 0.002 |
|  |  |  |  | - | Lobule X of vermis | 4.265 | 0.001 |
|  |  |  |  | Left | Red nucleus | 4.135 | 0.001 |
|  |  |  |  | Right | Red nucleus | 3.372 | 0.005 |
|  |  |  |  | Left | Substantia nigra | 3.150 | 0.008 |
|  |  |  |  | Right | Substantia nigra | 4.135 | 0.001 |

**Table S7** Significance and interpretation of each predictor on subsequent longitudinal degeneration

| **Measure** | **t/F Statistic** | ***P* Value** | **Relationship to Change** |
| --- | --- | --- | --- |
| Shortest path length to the top epicenter | 6.858 (F) | 1.02 × 10^-4^ | Higher, less degeneration (**Figure 5C_b**) |
| Euclidean distance to the top epicenter | 31.616 (F) | < 2 × 10^−16^ | Higher, less degeneration (**Figure 5C_c**) |
| Nodal hazard | 339.050 (F) | < 2 × 10^−16^ | Higher, more degeneration (**Figure 5C_d**) |
| UPDRS score | 0.442 (F) | 0.506 | Not significant |
| SPE * UPDRS | 7.941 (F) | 4.38 × 10^-7^ | Higher SPE-UPDRS, positive-shifted SPE curve |
| NH * UPDRS | 5.206 (F) | 1.52 × 10^-3^ | Higher NH-UPDRS, positive-shifted NH curve |
| Disease duration | 11.582 (F) | < 2 × 10^−16^ | Longer, more degeneration |
| Follow-up time | 53.452 (F) | < 2 × 10^−16^ | Longer, more degeneration |
| PD subtype | 9.507 (t) | < 2 × 10^−16^ | Different subtypes have different amounts of degeneration |
| Age | 5.498 (t) | 3.91× 10^-8^ | Higher, more degeneration |
| Sex | 0.730 (t) | 0.465 | Not significant |
| Global intercept | -3.463 (t) | 5.36 × 10^-4^ | Subjects’ degeneration on average |
| Cortical/subcortical node | -10.598 (t) | < 2 × 10^−16^ | Cortical, more degeneration |
| Subject intercept | 1.692 (F) | 0.085 | Not significant |
| Node random slope | 554.279 (F) | < 2 × 10^−16^ | Different nodes have different baseline/longitudinal degeneration relationships |
| Spatial autocorrelation | 1.799 (F) | 0.008 | Adjacent nodes have more similar amounts of change |

**Table S8** Characteristics of baseline, longitudinal, and predictive longitudinal significant degeneration regions in PD subtype 1

| **Baseline** | | | | **Longitudinal** | | | | **Predictive longitudinal** | | | |
| --- | --- | --- | --- | --- | --- | --- | --- | --- | --- | --- | --- |
| **Hemisphere** | **Region name** | **T values** | ***P*_FDR_ values** | **Hemisphere** | **Region name** | **T values** | ***P*_FDR_ values** | **Hemisphere** | **Region name** | **T values** | ***P*_FDR_ values** |
| Left | Lobule IX of cerebellar hemisphere | 3.777 | 0.031 | Left | Crus II of cerebellar hemisphere | 3.241 | 0.042 | Left | Crus II of cerebellar hemisphere | 3.990 | 0.007 |
| Left | Lobule X of cerebellar hemisphere | 4.088 | 0.024 | Right | Crus II of cerebellar hemisphere | 3.486 | 0.037 | Right | Crus II of cerebellar hemisphere | 4.196 | 0.004 |
|  |  |  |  | Left | Lobule III of cerebellar hemisphere | 3.356 | 0.039 | Left | Lobule III of cerebellar hemisphere | 3.753 | 0.010 |
|  |  |  |  | Left | Lobule VIII of cerebellar hemisphere | 3.766 | 0.021 | Left | Lobule IV, V of cerebellar hemisphere | 3.415 | 0.018 |
|  |  |  |  | Left | Lobule IX of cerebellar hemisphere | 3.953 | 0.018 | Left | Lobule VIII of cerebellar hemisphere | 4.193 | 0.004 |
|  |  |  |  | Right | Lobule IX of cerebellar hemisphere | 3.153 | 0.042 | Left | Lobule IX of cerebellar hemisphere | 5.818 | <0.001 |
|  |  |  |  | Left | Lobule X of cerebellar hemisphere | 4.038 | 0.018 | Right | Lobule IX of cerebellar hemisphere | 3.503 | 0.016 |
|  |  |  |  | - | Lobule I, II of vermis | 3.075 | 0.047 | Left | Lobule X of cerebellar hemisphere | 5.731 | <0.001 |
|  |  |  |  | - | Lobule IV, V of vermis | 3.168 | 0.042 | - | Lobule I, II of vermis | 3.274 | 0.025 |
|  |  |  |  | - | Lobule VII of vermis | 3.321 | 0.039 | - | Lobule III of vermis | 3.810 | 0.010 |
|  |  |  |  |  |  |  |  | - | Lobule IV, V of vermis | 3.659 | 0.011 |

**Table S9** Characteristics of baseline, longitudinal, and predictive longitudinal significant degeneration regions in PD subtype 2

| **Baseline** | | | | **Longitudinal** | | | | **Predictive longitudinal** | | | | |
| --- | --- | --- | --- | --- | --- | --- | --- | --- | --- | --- | --- | --- |
| **Hemisphere** | **Region name** | **T values** | ***P*_FDR_ values** | **Hemisphere** | **Region name** | **T values** | ***P*_FDR_ values** | **Hemisphere** | **Region name** | **T values** | ***P*_FDR_ values** |  |
| Left | Angular gyrus | 4.826 | <0.001 | Left | Angular gyrus | 5.123 | <0.001 | Left | Angular gyrus | 5.649 | <0.001 |  |
| Left | Anterior cingulate cortex, pregenual | 2.492 | 0.027 | Left | Anterior cingulate cortex, pregenual | 2.420 | 0.029 | Right | Angular gyrus | 2.223 | 0.045 |  |
| Right | Anterior cingulate cortex, pregenual | 3.421 | 0.003 | Right | Anterior cingulate cortex, pregenual | 3.972 | 0.001 | Left | Anterior cingulate cortex, pregenual | 3.642 | 0.002 |  |
| Left | Anterior cingulate cortex, subgenual | 2.458 | 0.029 | Left | Anterior cingulate cortex, subgenual | 2.693 | 0.016 | Right | Anterior cingulate cortex, pregenual | 4.470 | <0.001 |  |
| Right | Anterior cingulate cortex, subgenual | 3.507 | 0.003 | Right | Anterior cingulate cortex, subgenual | 3.767 | 0.001 | Left | Anterior cingulate cortex, subgenual | 3.430 | 0.003 |  |
| Left | Anterior orbital gyrus | 2.703 | 0.018 | Left | Anterior cingulate cortex, supracallosal | 2.227 | 0.042 | Right | Anterior cingulate cortex, subgenual | 4.415 | <0.001 |  |
| Right | Anterior orbital gyrus | 2.436 | 0.030 | Right | Anterior cingulate cortex, supracallosal | 2.592 | 0.020 | Left | Anterior cingulate cortex, supracallosal | 2.888 | 0.010 |  |
| Left | Calcarine fissure and surrounding cortex | 2.551 | 0.025 | Left | Anterior orbital gyrus | 3.812 | 0.001 | Right | Anterior cingulate cortex, supracallosal | 2.820 | 0.011 |  |
| Left | Cuneus | 3.190 | 0.006 | Right | Anterior orbital gyrus | 2.397 | 0.030 | Left | Anterior orbital gyrus | 3.814 | 0.001 |  |
| Right | Fusiform gyrus | 2.256 | 0.042 | Left | Calcarine fissure and surrounding cortex | 3.521 | 0.002 | Right | Anterior orbital gyrus | 3.349 | 0.003 |  |
| Left | Gyrus rectus | 3.123 | 0.007 | Left | Cuneus | 4.259 | <0.001 | Left | Calcarine fissure and surrounding cortex | 3.410 | 0.003 |  |
| Right | Gyrus rectus | 3.527 | 0.003 | Right | Cuneus | 2.698 | 0.016 | Left | Cuneus | 3.807 | 0.001 |  |
| Left | Heschl’s gyrus | 3.098 | 0.008 | Right | Fusiform gyrus | 2.831 | 0.012 | Right | Cuneus | 2.717 | 0.015 |  |
| Right | Inferior frontal gyrus, opercular part | 3.918 | 0.001 | Left | Gyrus rectus | 3.657 | 0.001 | Right | Fusiform gyrus | 2.852 | 0.011 |  |
| Left | Inferior frontal gyrus, pars orbitalis | 3.994 | 0.001 | Right | Gyrus rectus | 3.767 | 0.001 | Left | Gyrus rectus | 4.254 | <0.001 |  |
| Right | Inferior frontal gyrus, pars orbitalis | 4.427 | <0.001 | Left | Heschl’s gyrus | 3.030 | 0.008 | Right | Gyrus rectus | 4.788 | <0.001 |  |
| Left | Inferior frontal gyrus, triangular part | 3.524 | 0.003 | Right | Heschl’s gyrus | 2.166 | 0.048 | Left | Heschl’s gyrus | 3.534 | 0.002 |  |
| Right | Inferior frontal gyrus, triangular part | 4.466 | <0.001 | Left | Hippocampus | 2.885 | 0.010 | Right | Heschl’s gyrus | 2.374 | 0.033 |  |
| Left | Inferior parietal gyrus, excluding supramarginal and angular gyri | 3.259 | 0.005 | Right | Hippocampus | 2.158 | 0.049 | Left | Hippocampus | 2.322 | 0.036 |  |
| Right | Inferior parietal gyrus, excluding supramarginal and angular gyri | 6.993 | <0.001 | Left | Inferior frontal gyrus, opercular part | 2.805 | 0.012 | Left | Inferior frontal gyrus, opercular part | 2.975 | 0.008 |  |
| Left | Inferior temporal gyrus | 2.980 | 0.010 | Right | Inferior frontal gyrus, opercular part | 4.164 | <0.001 | Right | Inferior frontal gyrus, opercular part | 4.734 | <0.001 |  |
| Right | Inferior temporal gyrus | 4.650 | <0.001 | Left | Inferior frontal gyrus, pars orbitalis | 4.385 | <0.001 | Left | Inferior frontal gyrus, pars orbitalis | 4.693 | <0.001 |  |
| Left | Inferior occipital gyrus | 4.444 | <0.001 | Right | Inferior frontal gyrus, pars orbitalis | 4.917 | <0.001 | Right | Inferior frontal gyrus, pars orbitalis | 4.956 | <0.001 |  |
| Right | Inferior occipital gyrus | 2.238 | 0.043 | Left | Inferior frontal gyrus, triangular part | 3.208 | 0.005 | Left | Inferior frontal gyrus, triangular part | 4.297 | <0.001 |  |
| Left | Insula | 3.098 | 0.008 | Right | Inferior frontal gyrus, triangular part | 5.164 | <0.001 | Right | Inferior frontal gyrus, triangular part | 5.415 | <0.001 |  |
| Left | Lateral orbital gyrus | 3.181 | 0.006 | Left | Inferior parietal gyrus, excluding supramarginal and angular gyri | 3.341 | 0.003 | Left | Inferior occipital gyrus | 5.229 | <0.001 |  |
| Right | Lateral orbital gyrus | 2.438 | 0.030 | Right | Inferior parietal gyrus, excluding supramarginal and angular gyri | 6.610 | <0.001 | Right | Inferior occipital gyrus | 3.076 | 0.006 |  |
| Left | Medial orbital gyrus | 4.275 | 0.001 | Left | Inferior temporal gyrus | 3.696 | 0.001 | Left | Inferior parietal gyrus, excluding supramarginal and angular gyri | 3.896 | 0.001 |  |
| Right | Medial orbital gyrus | 4.465 | <0.001 | Right | Inferior temporal gyrus | 5.817 | <0.001 | Right | Inferior parietal gyrus, excluding supramarginal and angular gyri | 7.896 | <0.001 |  |
| Left | Middle cingulate & paracingulate gyri | 3.707 | 0.002 | Left | Insula | 3.020 | 0.008 | Left | Inferior temporal gyrus | 3.878 | 0.001 |  |
| Right | Middle cingulate & paracingulate gyri | 4.083 | 0.001 | Right | Insula | 3.028 | 0.008 | Right | Inferior temporal gyrus | 5.425 | <0.001 |  |
| Left | Middle frontal gyrus | 3.125 | 0.007 | Left | Lateral orbital gyrus | 3.939 | 0.001 | Left | Insula | 4.410 | <0.001 |  |
| Right | Middle frontal gyrus | 2.902 | 0.011 | Left | Lingual gyrus | 2.552 | 0.022 | Right | Insula | 2.955 | 0.008 |  |
| Left | Middle occipital gyrus | 3.788 | 0.001 | Right | Lingual gyrus | 2.449 | 0.027 | Left | Lateral orbital gyrus | 4.219 | <0.001 |  |
| Right | Middle occipital gyrus | 2.500 | 0.027 | Left | Medial orbital gyrus | 4.871 | <0.001 | Right | Lateral orbital gyrus | 3.311 | 0.003 |  |
| Left | Middle temporal gyrus | 4.205 | 0.001 | Right | Medial orbital gyrus | 5.415 | <0.001 | Left | Medial orbital gyrus | 5.261 | <0.001 |  |
| Right | Middle temporal gyrus | 4.384 | <0.001 | Left | Middle cingulate & paracingulate gyri | 4.069 | 0.001 | Right | Medial orbital gyrus | 5.171 | <0.001 |  |
| Left | Olfactory cortex | 5.470 | <0.001 | Right | Middle cingulate & paracingulate gyri | 4.240 | <0.001 | Left | Middle cingulate & paracingulate gyri | 4.605 | <0.001 |  |
| Right | Olfactory cortex | 5.197 | <0.001 | Left | Middle frontal gyrus | 4.165 | <0.001 | Right | Middle cingulate & paracingulate gyri | 4.847 | <0.001 |  |
| Right | Parahippocampal gyrus | 3.997 | 0.001 | Right | Middle frontal gyrus | 3.683 | 0.001 | Left | Middle frontal gyrus | 3.807 | 0.001 |  |
| Left | Postcentral gyrus | 4.453 | <0.001 | Left | Middle occipital gyrus | 4.625 | <0.001 | Right | Middle frontal gyrus | 3.534 | 0.002 |  |
| Right | Postcentral gyrus | 5.792 | <0.001 | Right | Middle occipital gyrus | 2.919 | 0.010 | Left | Middle occipital gyrus | 4.315 | <0.001 |  |
| Left | Posterior cingulate gyrus | 3.666 | 0.002 | Left | Middle temporal gyrus | 4.851 | <0.001 | Right | Middle occipital gyrus | 3.154 | 0.005 |  |
| Right | Posterior cingulate gyrus | 2.587 | 0.024 | Right | Middle temporal gyrus | 5.214 | <0.001 | Left | Middle temporal gyrus | 4.796 | <0.001 |  |
| Left | Posterior orbital gyrus | 3.415 | 0.003 | Left | Olfactory cortex | 6.118 | <0.001 | Right | Middle temporal gyrus | 5.157 | <0.001 |  |
| Right | Posterior orbital gyrus | 3.546 | 0.003 | Right | Olfactory cortex | 6.133 | <0.001 | Left | Olfactory cortex | 6.353 | <0.001 |  |
| Left | Precentral gyrus | 2.584 | 0.024 | Left | Paracentral lobule | 2.910 | 0.010 | Right | Olfactory cortex | 6.058 | <0.001 |  |
| Left | Precuneus | 6.588 | <0.001 | Left | Parahippocampal gyrus | 2.863 | 0.011 | Left | Paracentral lobule | 2.188 | 0.048 |  |
| Right | Precuneus | 3.869 | 0.001 | Right | Parahippocampal gyrus | 4.486 | <0.001 | Left | Parahippocampal gyrus | 2.581 | 0.020 |  |
| Left | Red nucleus | 2.318 | 0.037 | Left | Postcentral gyrus | 5.004 | <0.001 | Right | Parahippocampal gyrus | 5.106 | <0.001 |  |
| Right | Red nucleus | 2.293 | 0.039 | Right | Postcentral gyrus | 5.106 | <0.001 | Left | Posterior cingulate gyrus | 4.787 | <0.001 |  |
| Right | Rolandic operculum | 3.698 | 0.002 | Left | Posterior cingulate gyrus | 4.526 | <0.001 | Right | Posterior cingulate gyrus | 3.601 | 0.002 |  |
| Left | Substantia nigra | 2.540 | 0.025 | Right | Posterior cingulate gyrus | 3.824 | 0.001 | Left | Posterior orbital gyrus | 4.169 | <0.001 |  |
| Right | Substantia nigra | 2.899 | 0.011 | Left | Posterior orbital gyrus | 4.540 | <0.001 | Right | Posterior orbital gyrus | 4.409 | <0.001 |  |
| Left | Superior frontal gyrus, dorsolateral | 3.988 | 0.001 | Right | Posterior orbital gyrus | 3.914 | 0.001 | Left | Postcentral gyrus | 5.066 | <0.001 |  |
| Right | Superior frontal gyrus, dorsolateral | 4.754 | <0.001 | Left | Precentral gyrus | 3.717 | 0.001 | Right | Postcentral gyrus | 6.564 | <0.001 |  |
| Left | Superior frontal gyrus, medial | 2.476 | 0.028 | Left | Precuneus | 6.677 | <0.001 | Left | Precentral gyrus | 3.223 | 0.004 |  |
| Right | Superior frontal gyrus, medial | 2.342 | 0.036 | Right | Precuneus | 4.899 | <0.001 | Left | Precuneus | 7.373 | <0.001 |  |
| Left | Superior frontal gyrus, medial orbital | 2.367 | 0.034 | Left | Rolandic operculum | 2.280 | 0.038 | Right | Precuneus | 4.607 | <0.001 |  |
| Right | Superior frontal gyrus, medial orbital | 3.589 | 0.002 | Right | Rolandic operculum | 4.012 | 0.001 | Left | Rolandic operculum | 2.574 | 0.020 |  |
| Left | Superior occipital gyrus | 3.824 | 0.001 | Right | Substantia nigra | 2.416 | 0.029 | Right | Rolandic operculum | 4.392 | <0.001 |  |
| Left | Superior parietal gyrus | 4.060 | 0.001 | Left | Superior frontal gyrus, dorsolateral | 4.281 | <0.001 | Left | Superior frontal gyrus, dorsolateral | 4.743 | <0.001 |  |
| Right | Superior parietal gyrus | 4.225 | 0.001 | Right | Superior frontal gyrus, dorsolateral | 5.541 | <0.001 | Right | Superior frontal gyrus, dorsolateral | 5.628 | <0.001 |  |
| Left | Superior temporal gyrus | 3.023 | 0.009 | Left | Superior frontal gyrus, medial | 3.707 | 0.001 | Left | Superior frontal gyrus, medial | 3.403 | 0.003 |  |
| Right | Superior temporal gyrus | 4.046 | 0.001 | Right | Superior frontal gyrus, medial | 2.591 | 0.020 | Right | Superior frontal gyrus, medial | 3.281 | 0.004 |  |
| Left | SupraMarginal gyrus | 4.631 | <0.001 | Left | Superior frontal gyrus, medial orbital | 2.666 | 0.017 | Left | Superior frontal gyrus, medial orbital | 3.323 | 0.003 |  |
| Right | SupraMarginal gyrus | 5.276 | <0.001 | Right | Superior frontal gyrus, medial orbital | 3.800 | 0.001 | Right | Superior frontal gyrus, medial orbital | 4.397 | <0.001 |  |
| Left | Temporal pole: middle temporal gyrus | 3.023 | 0.009 | Left | Superior occipital gyrus | 4.217 | <0.001 | Left | Superior occipital gyrus | 4.550 | <0.001 |  |
| Right | Temporal pole: middle temporal gyrus | 2.275 | 0.040 | Left | Superior parietal gyrus | 4.327 | <0.001 | Right | Superior occipital gyrus | 2.312 | 0.037 |  |
| Left | Temporal pole: superior temporal gyrus | 2.377 | 0.034 | Right | Superior parietal gyrus | 4.301 | <0.001 | Left | Superior parietal gyrus | 4.907 | <0.001 |  |
| Left | Thalamus | 3.626 | 0.002 | Left | Superior temporal gyrus | 3.479 | 0.002 | Right | Superior parietal gyrus | 5.077 | <0.001 |  |
| Right | Thalamus | 4.996 | <0.001 | Right | Superior temporal gyrus | 3.670 | 0.001 | Left | Superior temporal gyrus | 3.843 | 0.001 |  |
|  |  |  |  | Left | Supplementary motor area | 2.230 | 0.042 | Right | Superior temporal gyrus | 4.674 | <0.001 |  |
|  |  |  |  | Right | Supplementary motor area | 2.318 | 0.035 | Left | SupraMarginal gyrus | 5.168 | <0.001 |  |
|  |  |  |  | Left | SupraMarginal gyrus | 4.411 | <0.001 | Right | SupraMarginal gyrus | 6.249 | <0.001 |  |
|  |  |  |  | Right | SupraMarginal gyrus | 5.155 | <0.001 | Left | Temporal pole: middle temporal gyrus | 4.372 | <0.001 |  |
|  |  |  |  | Left | Temporal pole: middle temporal gyrus | 3.318 | 0.004 | Right | Temporal pole: middle temporal gyrus | 3.384 | 0.003 |  |
|  |  |  |  | Right | Temporal pole: middle temporal gyrus | 3.439 | 0.003 | Left | Temporal pole: superior temporal gyrus | 3.065 | 0.006 |  |
|  |  |  |  | Left | Temporal pole: superior temporal gyrus | 4.036 | 0.001 | Left | Thalamus | 2.598 | 0.020 |  |
|  |  |  |  | Left | Thalamus | 3.830 | 0.001 | Right | Thalamus | 3.959 | 0.001 |  |
|  |  |  |  | Right | Thalamus | 3.867 | 0.001 |  |  |  |  |  |

# Supplemental Figures

**
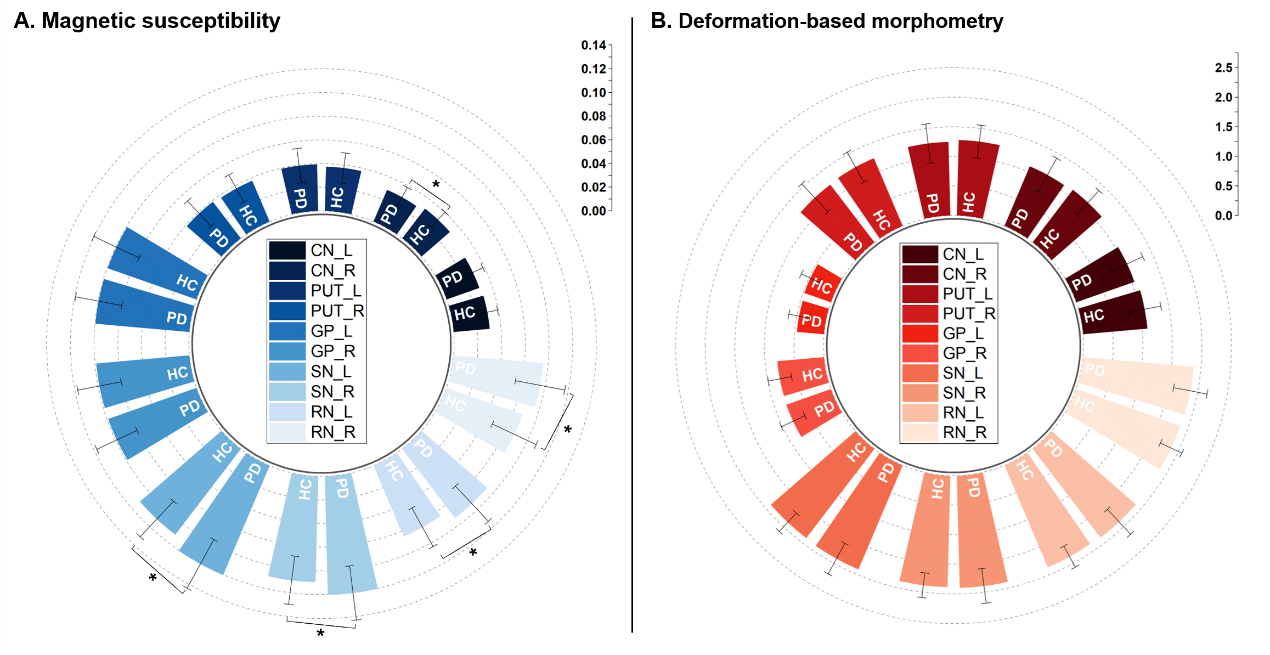
**

**Figure S1 Comparison of magnetic susceptibility and volume deformation in DBN between PD and HC.** Generalized linear models were used to assess magnetic susceptibility differences between PD patients and HC, adjusted for age and sex **(A)**;

volume deformation differences were compared between PD and HC, adjusted for age, sex, and total intracranial volume **(B)**. False discovery rate correction was applied for multiple comparisons. *Indicates statistically significant differences.

CN = caudate; DBN = deep brain nuclei; GP = globus pallidus; HC = healthy controls; L = left; PD = Parkinson's disease; PUT = putamen; RN = red nucleus; R= right; SN = substantia nigra.


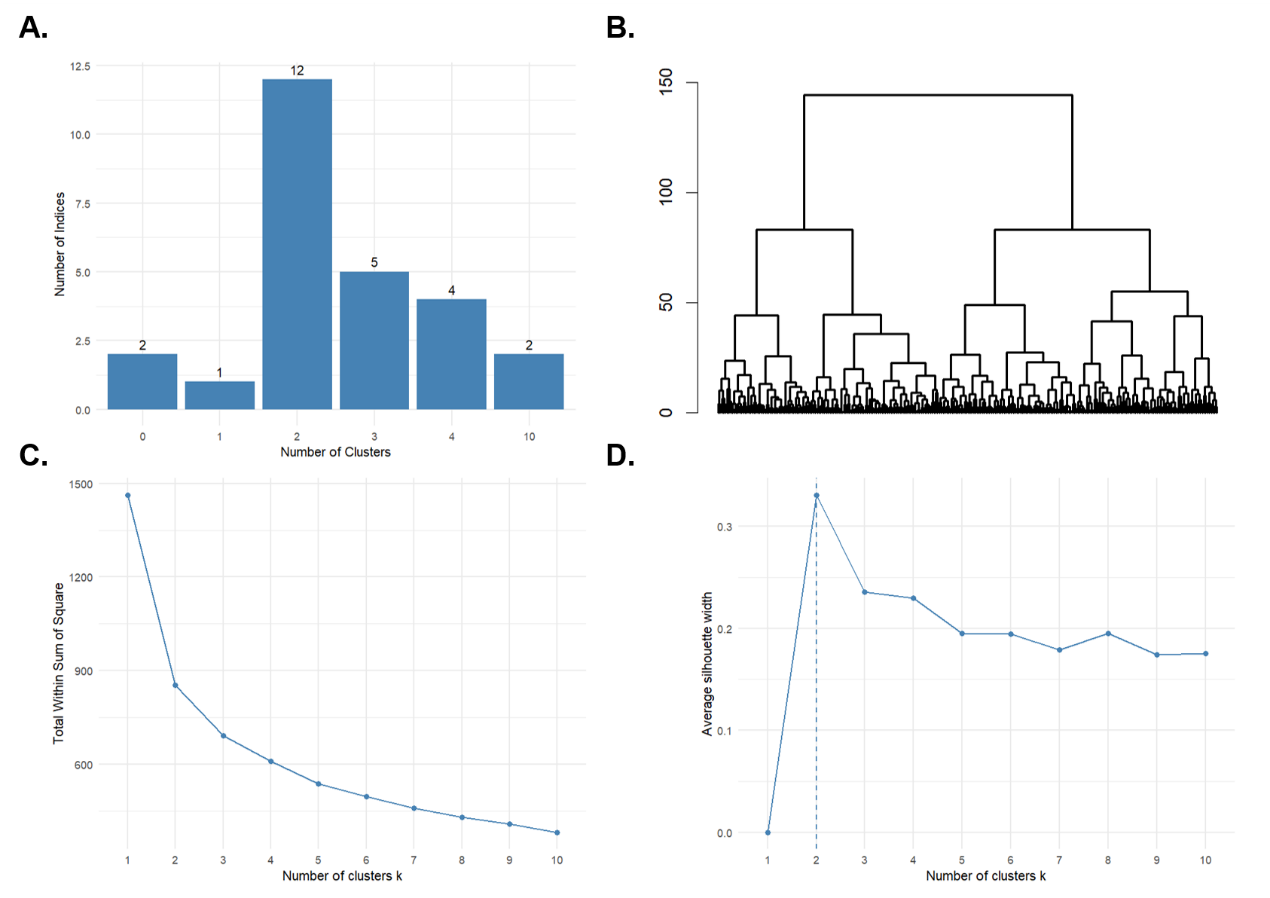


**Figure S2** **Determination of the optimal cluster number and hierarchical clustering. A.** Histogram of NbClust indices, with most indices supporting a two-cluster solution. **B.** Hierarchical clustering dendrogram using Ward’s method; leaf labels are omitted for clarity. **C.** Elbow plot of the total within-cluster sum of squares for k = 1–10, showing an inflection point at k = 2, suggesting it as the optimal number of clusters. **D.** Silhouette plot for k = 2–10, with maximum average silhouette width at k = 2, confirming two clusters as optimal.


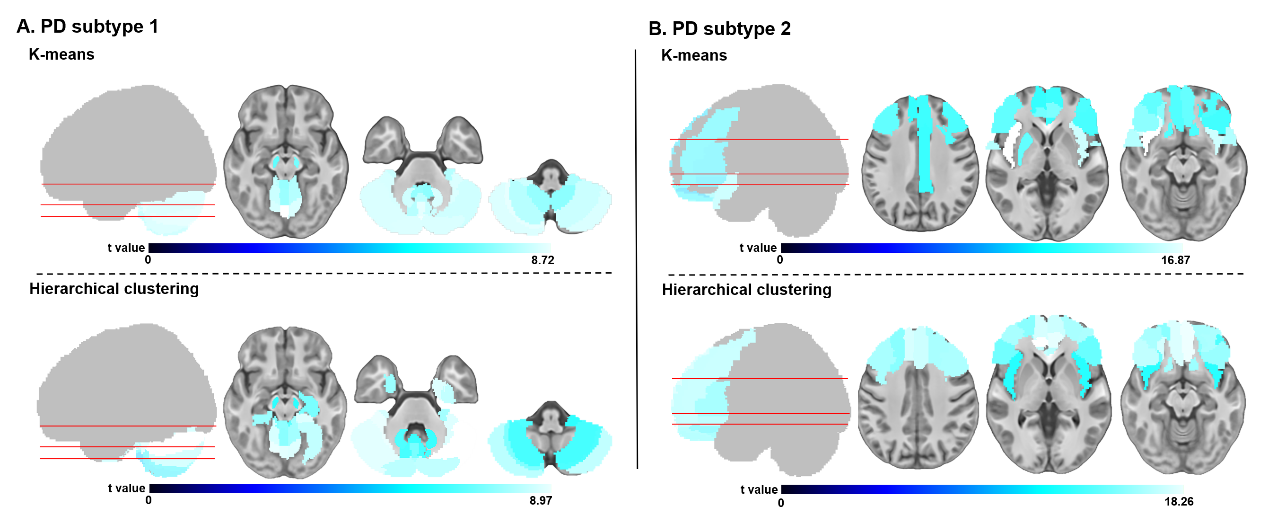


**Figure S3. Top 30 epicenter distribution of PD subtypes using different clustering methods.** Distribution of the top 30 epicenter regions for two PD subtypes (A: subtype 1; B: subtype 2) identified using K-means (first row; n_subtype 1_ = 142, n_subtype 2_ = 136) and hierarchical clustering (second row; n_subtype 1_ = 156, n_subtype 2_ = 122). The color bar indicates t-values from one-sample t-tests of epicenter goodness-of-fit scores in each region, with lighter colors representing higher t-values and greater epicenter degree.

FDR = false discovery rate; PD = Parkinson’s disease.

**
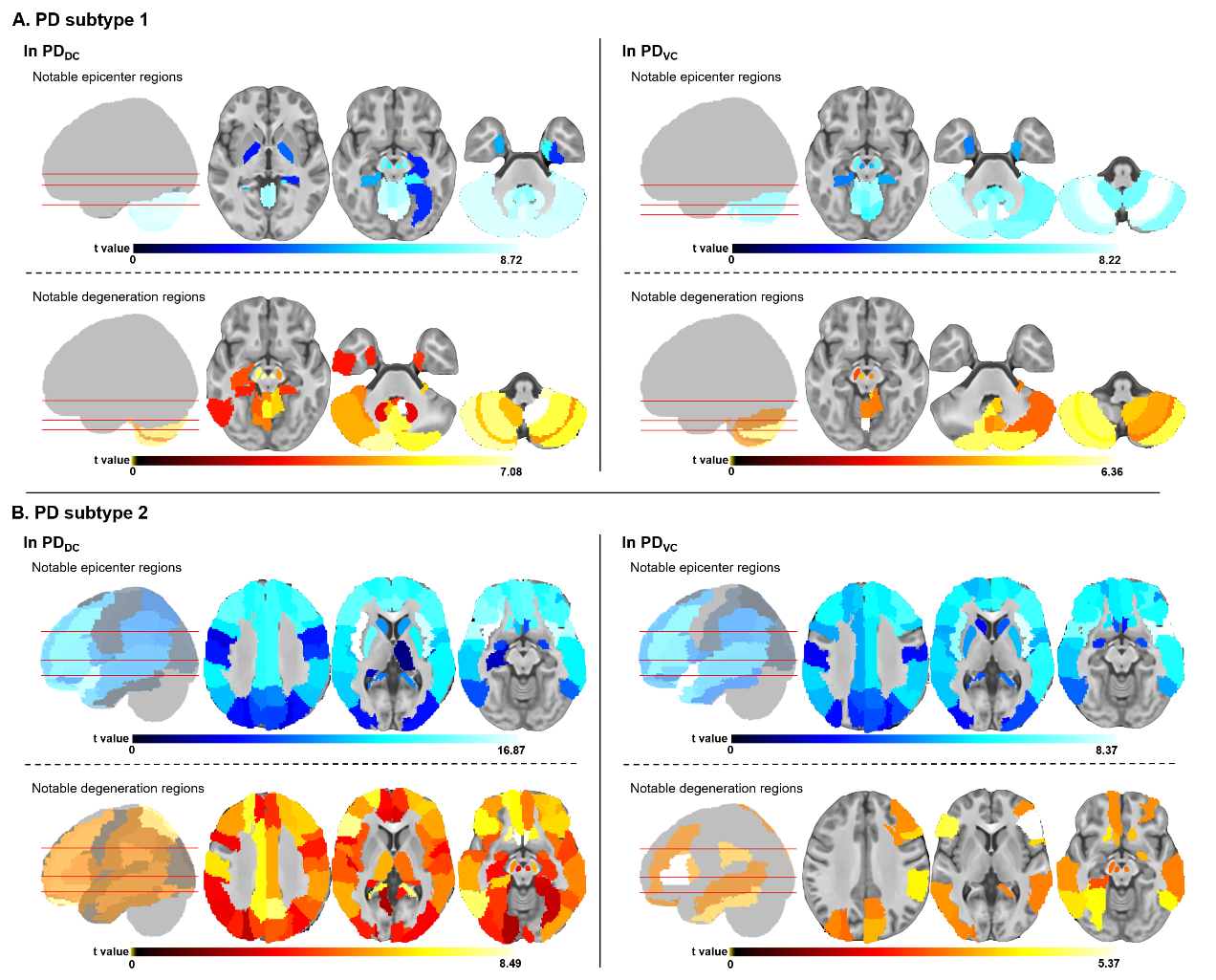
**

**Figure S4 Epicenter distribution and brain degeneration map of PD subtypes.** Notable epicenter (bule) and degeneration (red) regions for the two PD subtypes (A: subtype 1, B: subtype 2) in PD_DC_ and PD_VC_ (FDR *P* < 0.05). The color bar of the t-value indicates the magnitude of epicenter goodness-of-fit score or degeneration z-score in each region, with lighter colors representing higher values.

FDR = false discovery rate; PD = Parkinson’s disease; PD_DC_ = PD discovery cohort; PD_VC_ = PD validation cohort.


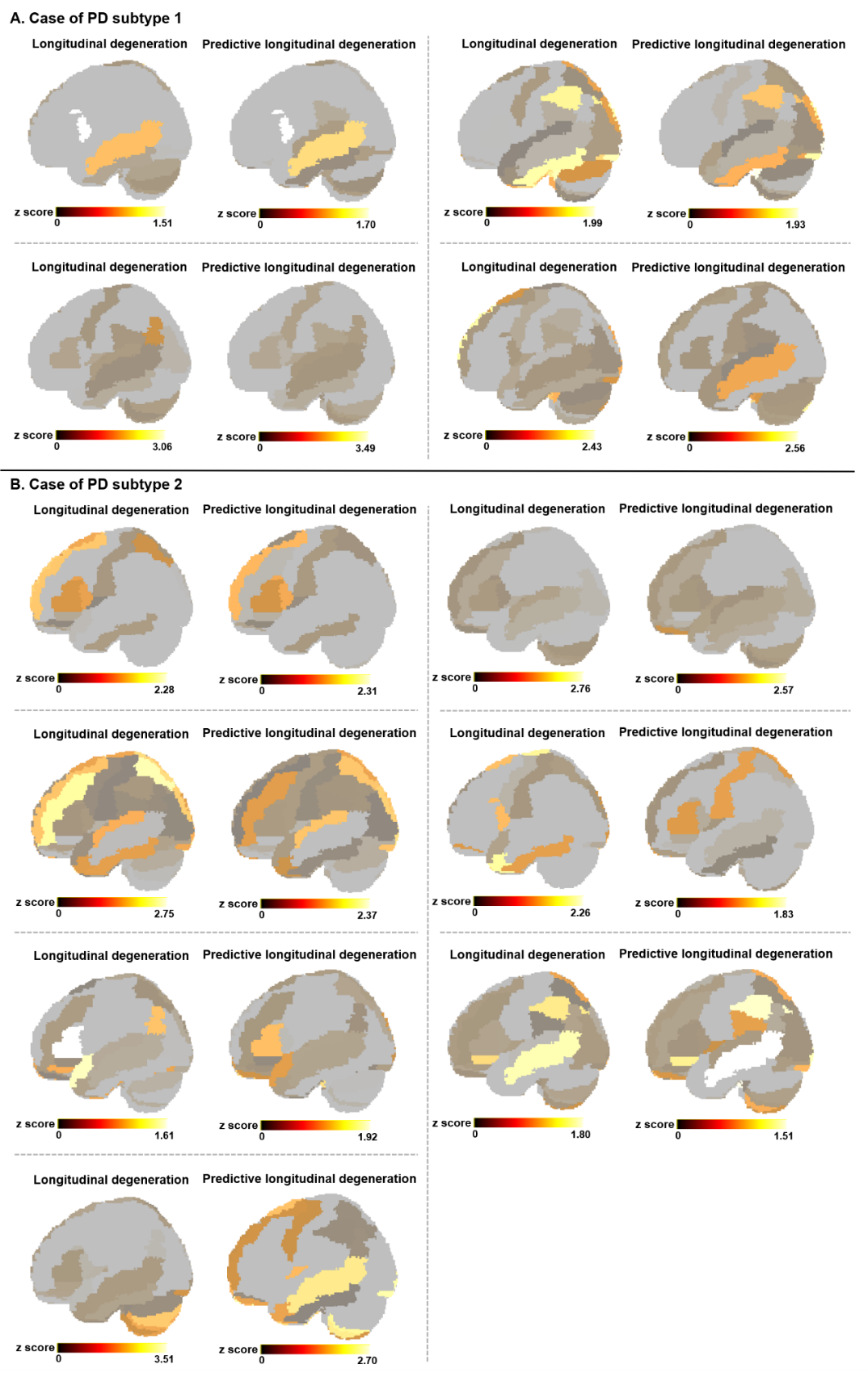


**Figure S5** **Actual and predictive longitudinal degeneration mapping of patients in validation cohort.** Actual and predictive longitudinal degeneration z-score maps for all patients in validation cohort are shown (z-score > 0). The color bar of z-score indicates the degeneration degree of each region, with lighter colors representing higher scores.

PD = Parkinson’s disease.
